# Comparison of the Effects of Four Laser Wavelengths on Medication-Related Osteonecrosis of the Jaw (MRONJ) in a Murine Model: An *In Vivo* Photobiomodulation Study

**DOI:** 10.7150/ijms.93224

**Published:** 2024-11-11

**Authors:** Mustafa Ayhan, Betul Gedik, Ekrem Emir Kalelioglu, Abdulsamet Kundakcioglu, Canan Kucukgergin, Cevat Tugrul Turgut, Humeyra KOCAELLI, Fatma Canan Alatli, Halim Issever, Evin Ademoglu, Mehmet YALTIRIK

**Affiliations:** 1 Istanbul University Faculty of Dentistry Department of Oral and Maxillofacial Surgery, Prof. Dr. Cavit Orhan Tutengil Street No. 4 Vezneciler Fatih, Istanbul, Turkey.; 2 Istanbul University Faculty of Medicine Department of Medical Biochemistry, Istanbul Tıp Fakültesi Çapa Fatih, İstanbul, Turkey.; 3 Istanbul Health and Technology University Department of Medical Pathology, Sütlüce İmrahor Caddesi No. 82 Beyoğlu, İstabul, Turkey.; 4 Istanbul University Faculty of Medicine, Department of Internal Medicine, Department of Public Health Istanbul Tıp Fakültesi Çapa Fatih, İstanbul, Turkey.

**Keywords:** Photobiomodulation therapy, Laser therapy, Medication-related osteonecrosis of the jaw, Zoledronate, Vitamin D, Dentistry, Oral surgery

## Abstract

**Background:** This study aims to investigate the effectiveness of lasers at various wavelengths in treating medication-related osteonecrosis of the jaw (MRONJ) using biochemical, clinical scoring, micro CT analysis, and histopathological methods. The study follows the ARRIVE guidelines to ensure robust and transparent research.

**Methods:** In our study, there were 6 groups, including one SHAM group, one CONTROL group, and four experimental groups, with 8 rats in each individual group. Each rat received antiresorptive drug intraperitoneally for 4 weeks and then had the left second molar in the mandible extracted. All animals were sacrificed at the end of the 12th week. In the experimental groups, lasers at wavelengths of 405nm, 445nm, 660nm, and 808nm were applied to the animals. Parameters such as serum vitamin D levels, bone density and bone volume at the extraction site, new bone formation, dead bone count, inflammatory cell count, and epithelial regeneration were examined. Additionally, clinical scoring was conducted after sacrifice. The laser parameters included power density, area, time, fluence, and mode (continuous wave), and the light was administered using a fiber with a Gaussian profile. Statistical analyses were performed with the NCSS (Number Cruncher Statistical System) 2007 Statistical Software (Utah, USA) package program. The results were evaluated at the p<0.05 significance level.

**Results:** According to the results obtained from our study, new bone formation in all experimental groups was significantly higher than in the SHAM and CONTROL groups. Furthermore, the 660nm and 808nm wavelengths increased serum vitamin D levels significantly. The most successful outcomes were observed in clinical scoring, dead bone count, epithelial cell regeneration, and bone density in the 660nm and 808nm wavelength groups.

**Conclusions:** The combined use of lasers at 660nm and 808nm wavelengths may yield successful results in treating MRONJ.

## Introduction

Medication-related osteonecrosis of the jaws (MRONJ) represents a severe adverse drug reaction characterized by progressive bone deterioration affecting the maxilla and mandible in patients undergoing treatment for conditions such as osteoporosis, osteopenia, cancer, hypercalcemia of malignancy, bone metastases from solid tumors, or lytic lesions associated with multiple myeloma. The primary culprits responsible for MRONJ are antiresorptive agents, encompassing both nitrogen-containing and non-containing bisphosphonates (BPs), as well as receptor activators of nuclear factor kappa-B ligand (RANK-L) inhibitors. Additionally, antiangiogenic agents like bevacizumab, sunitinib, and sorafenib have been implicated in MRONJ development 1-5.

While the exact pathophysiology of MRONJ remains not fully understood, it is postulated that a combination of factors contributes to its onset. These factors may include inflammation or infection, invasive dental procedures involving bone such as tooth extraction or minor trauma, compromised immunity, vitamin D deficiency, excessive inhibition of bone resorption, angiogenesis suppression, and altered bone remodeling 6-10.

MRONJ usually clinically presents with gingival ulcers in the region of exposed bone. As the disease progresses, it leads to severe pain, purulent drainage, intraoral or extraoral fistula, impaired oroantral communication, and pathologic bone fracture 6. Several procedures on MRONJ management have been performed since its first description in 2003 6, 11, 12. However, the treatment strategy needs to be revised, with no consensus regarding non-surgical versus surgical approaches. Non-surgical management of MRONJ includes optimization of oral health by reinforcement of oral hygiene with regular brushing and antimicrobial mouth rinse, frequent dental checks, and antibiotic therapy. In contrast, surgical management includes removing necrotic bone area(s) 12.

Recent research results have emphasized adjuvant therapeutic approaches, including hyperbaric oxygen, photobiomodulation (previously known as low-level laser therapy or LLLT), and platelet-rich plasma 9, 13. These investigations have illuminated the potential advantages of incorporating photobiomodulation to complement conventional medical treatments, particularly in the early stages of MRONJ. Photobiomodulation exhibits superiority over antimicrobial mouthwashes and antibiotic therapy in these initial stages due to its capacity to modulate cellular metabolism, enhance wound healing processes, and alleviate pain 2, 7, 14-16. Conversely, for patients in advanced stages of MRONJ, minimally invasive surgical interventions, such as laser surgery, emerge as a more favorable alternative than traditional surgical approaches. The positive impact of laser irradiation on MRONJ treatment is attributed to its bio-stimulatory effects on the organic bone matrix and osteoblasts, including elevating the mitotic index and stimulating osteoblastic cell proliferation, differentiation, and activity 1, 6, 14-16.

Laser biostimulation (or biomodulation) has many applications, primarily in wound healing and pain management. Given the current lack of evidence supporting the use of laser-assisted interventions in MRONJ, we examined the effects of different wavelength diode lasers on soft and hard tissue healing in an experimental MRONJ model in rats based on evaluations of biochemical markers, radiological findings, clinical assessment, and histopathological findings 16-18.

## Materials and Methods

### Animal Handling

A total of 48 male Sprague-Dawley rats (Aziz Sancar Institute of Experimental Medicine, Istanbul, Turkey), 12 weeks old and weighing 250±20 g, were used in the study. Sample size was calculated using a power analysis based on preliminary data, aiming for a power of 80% and an alpha level of 0.05. The rats were housed in a temperature, humidity, and light-controlled room (23±3 °C; 55±15%; 12h light, dark cycle); standard laboratory chow and tap water were supplied ad libitum. The experimental protocol of this study was reviewed and approved by the Istanbul University Animal Experiments Local Ethics Committee (approval code: 205272). All methods are reported per ARRIVE guidelines (https://arriveguidelines.org/arrive-guidelines) 18. The completed ARRIVE author checklist is included as supplementary material.

### Study Design and Surgical Procedure

MRONJ formation process in rats: After all animals adapted to the laboratory conditions, 8 rats were randomly assigned to a sham group. The remaining 40 rats received 0.06 mg/kg zoledronic acid (ZOL, Zometa, Novartis, Basel, Switzerland) diluted in phosphate-buffered saline, pH 7.4, via intraperitoneal injections. Weekly doses were administered for 4 weeks to develop bisphosphonate-related jaw osteonecrosis. Following four ZOL injections, the rats were anesthetized with 0.5 mL/kg intramuscular injection of ketamine (100 mg/mL) (Ketalar, Pfizer, New York, USA). The mandibular left first molars of all animals were extracted. After cleaning the wound surfaces with polyvinylpyrrolidone (PVP) antiseptic solution (Povidone-Iodine, Betadine, Purdue Products, Stamford, USA), a single dose of tramadol (2 mg/kg) (Contramal, Abdi Ibrahim, Istanbul, Turkey) and cefazolin (50 mg/kg) (Cefazolin Sodium, Akorn, Lake Forest, USA) were administered for two days postoperatively to prevent infection and pain. The sham group underwent a similar procedure, receiving only saline injections and mandibular first molar extraction 19 (Table 1).

### Laser Procedure

The rats were randomly divided into five groups: a control and 4 experimental groups (8 rats each). No photobiomodulation was applied to the rats in the control and sham groups. Photobiomodulation at different wavelengths was performed twice a week for 16 seconds for four weeks to a single standard point created in the socket of the extracted tooth with the laser tip positioned parallel to the long axis using the following irradiation parameters: 480 mW/cm² optical power intensity, 6 J/cm² irradiation dose, and 120 mW power in continuous operation mode. Consequently, 48 J/cm² energy was applied per rat by performing 1.5 J/point photobiomodulation for 16 seconds in 8 sessions. Photobiomodulation applications were performed on experimental group rats as follows: 405 nm for Group 1, 445 nm for Group 2, 660 nm for Group 3, and 808 nm for Group 4.

InGaN (Indium Gallium Nitride)-based diode laser for 405 nm/445 nm applications, AlGaInP (Aluminium Gallium Indium Phosphide)-based diode laser for 660 nm application, GaAlAs (Gallium Aluminium Arsenide)-based diode laser for 808 nm application were used with a spot size of 0.25 cm² (Kale CNC Ltd., Istanbul, Turkey). Full technical specifications of the lasers used are given in Table 2. The choice of wavelengths and power for photobiomodulation was guided by the aim to simulate optimal therapeutic conditions without tissue damage. Previous studies have shown that effective photobiomodulation typically requires a minimum power output of 105 mW to achieve therapeutic effects, while avoiding damage 20, 21. Hence, a power of 120 mW was selected for this study to ensure effective treatment while minimizing potential thermal damage.

The consistency of the power output was verified with a power meter (Model PM100, Thorlabs, Newton, NJ, USA) to ensure the power set on the instrument matched the actual irradiated power.

### Biochemical Analysis

At the end of the 12th week, rats were anesthetized with ether, and heart blood samples were taken and placed in yellow-capped gel tubes (BD Vacutainer, Franklin Lakes, USA). The samples were centrifuged at 1500 g for 10 minutes, and serum samples were stored at -80°C until analysis. Vitamin D levels in these samples were measured using a Biotech brand µQuant model ELISA system at Istanbul Medical Faculty Biochemistry Department Research Laboratory with a commercially available ELISA kit (USCN Life Science Inc, Wuhan, China), following the manufacturer's instructions.

### Sacrification

Following blood collections for biochemical analysis at the end of the 12th week of the study, all animals were anesthetized using intraperitoneal ketamine hydrochloride (50 mg/kg) (Ketalar, Pfizer, New York, USA) and xylazine (10 mg/kg) (Rompun, Bayer, Leverkusen, Germany), then a sacrification process was carried out.

### Clinical Evaluation

**Figure 1:**
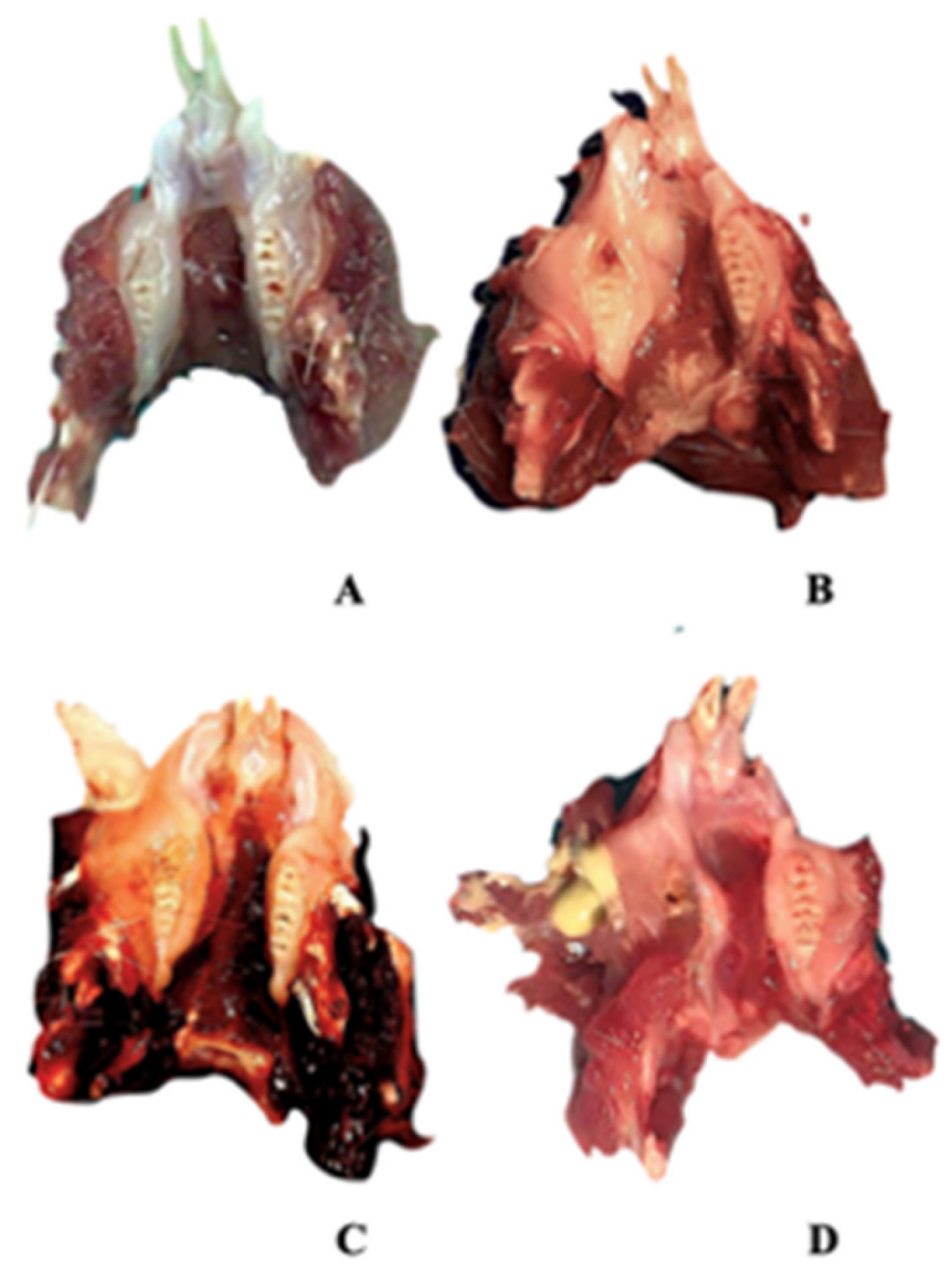
Clinical findings. An Empty well, healed socket of the extracted teeth B Hyperemic mucosa around the extraction site C Extraction site with impaired soft tissue integrity and bone exposure D Abscess and suppuration.

After the animals were sacrificed, their mandibles were dissected. They were all photographed for macroscopic examination and subjected to clinical scoring. In clinical scoring, 0 points for well-healed sockets, 1 point for hyperemic mucosa around the extraction site, 2 points for impaired soft tissue integrity and exposed bone, and 3 points for the presence of abscess and suppuration were given (Figure 1). Two blinded observers conducted the entire clinical assessments according to these criteria.

### Bone Scan Analysis with Micro Computed Tomography (Micro Ct)

**Figure 2:**
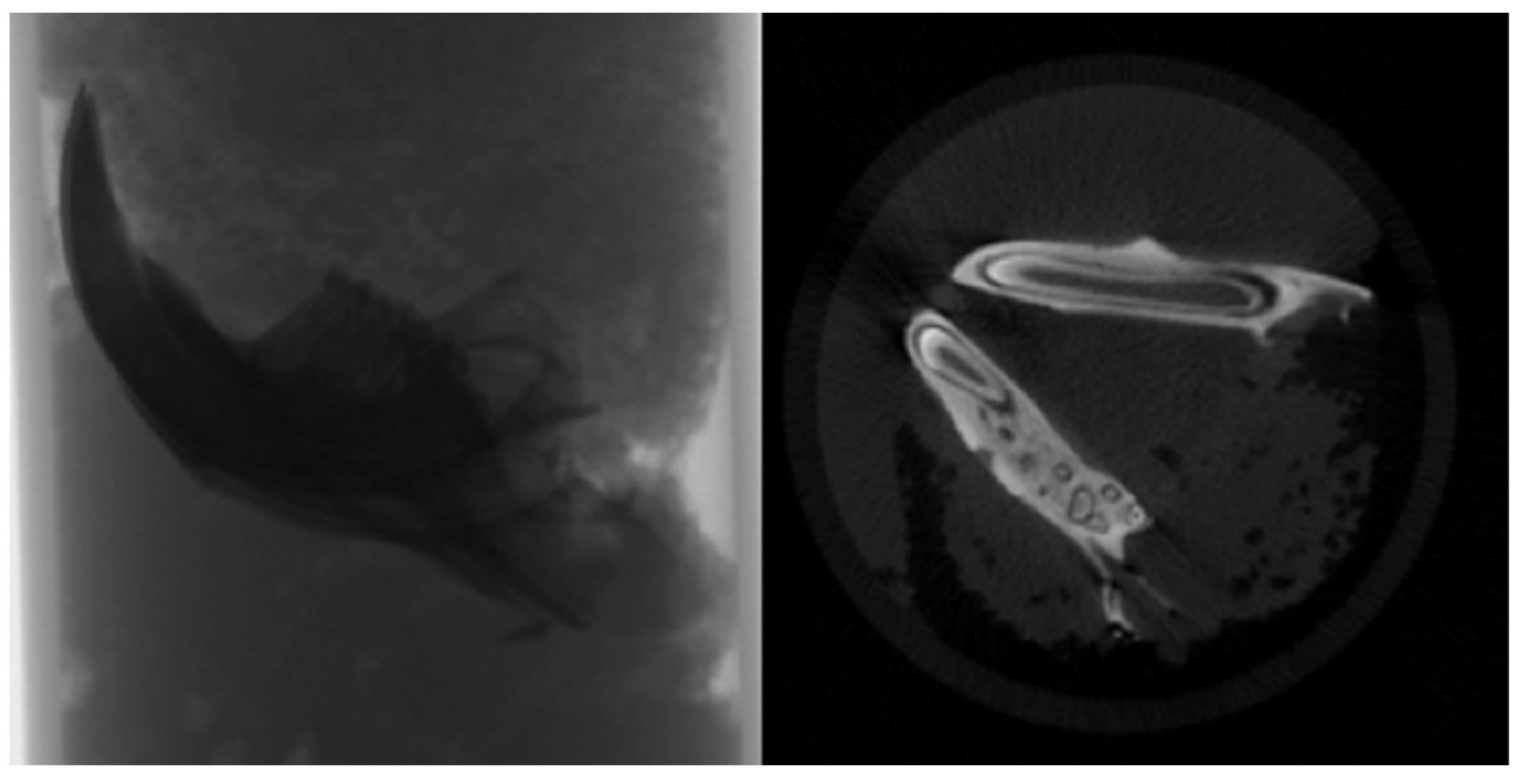
Raw image and sectional view.

The specimens were meticulously prepared for scanning in a SkyScan 1174v2 computed tomography apparatus located in Kontich, Belgium (Bruker, Kontich, Belgium). Each sample underwent scanning with a 0.25 mm aluminum filter, utilizing a voltage of 50 kVp, a current of 800 μA, and a power setting of 40 W. The scan parameters were finely tuned to optimize an exposure time of 3500 ms, achieve a resolution of 1024x1304 pixels, and employ a zoom factor of 30.51 μm. Each sample underwent a complete 360-degree rotation with angular increments of 1.00 degrees, requiring approximately 50 minutes for completion (Figure 2).

Following the scanning procedure, a total of 361 raw images were generated in TIFF format, and these images were subjected to reconstruction utilizing the NRecon software (Version 1.6.10.2) (Bruker, Kontich, Belgium). Subsequently, approximately 800 sections were extracted in BMP format, as depicted in Figure 2. These sections were imported into the CTan software (Version 1.16.4.1+) (Bruker, Kontich, Belgium), facilitating the execution of densitometric and morphometric measurements to establish quantitative parameters and create visual models.

**Figure 3:**
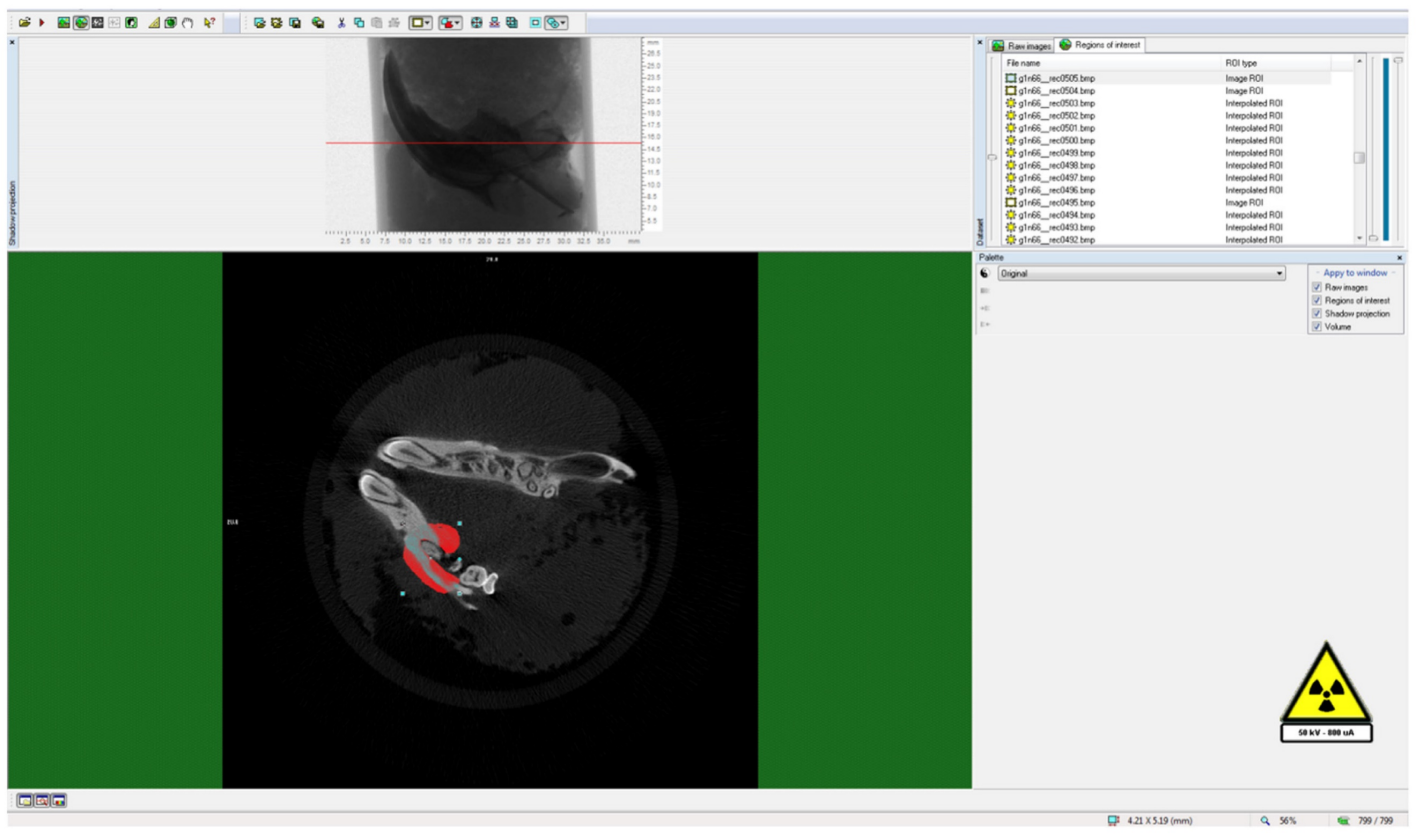
Determination of ROI (Region of Interest) around the analysis area.

**Figure 4:**
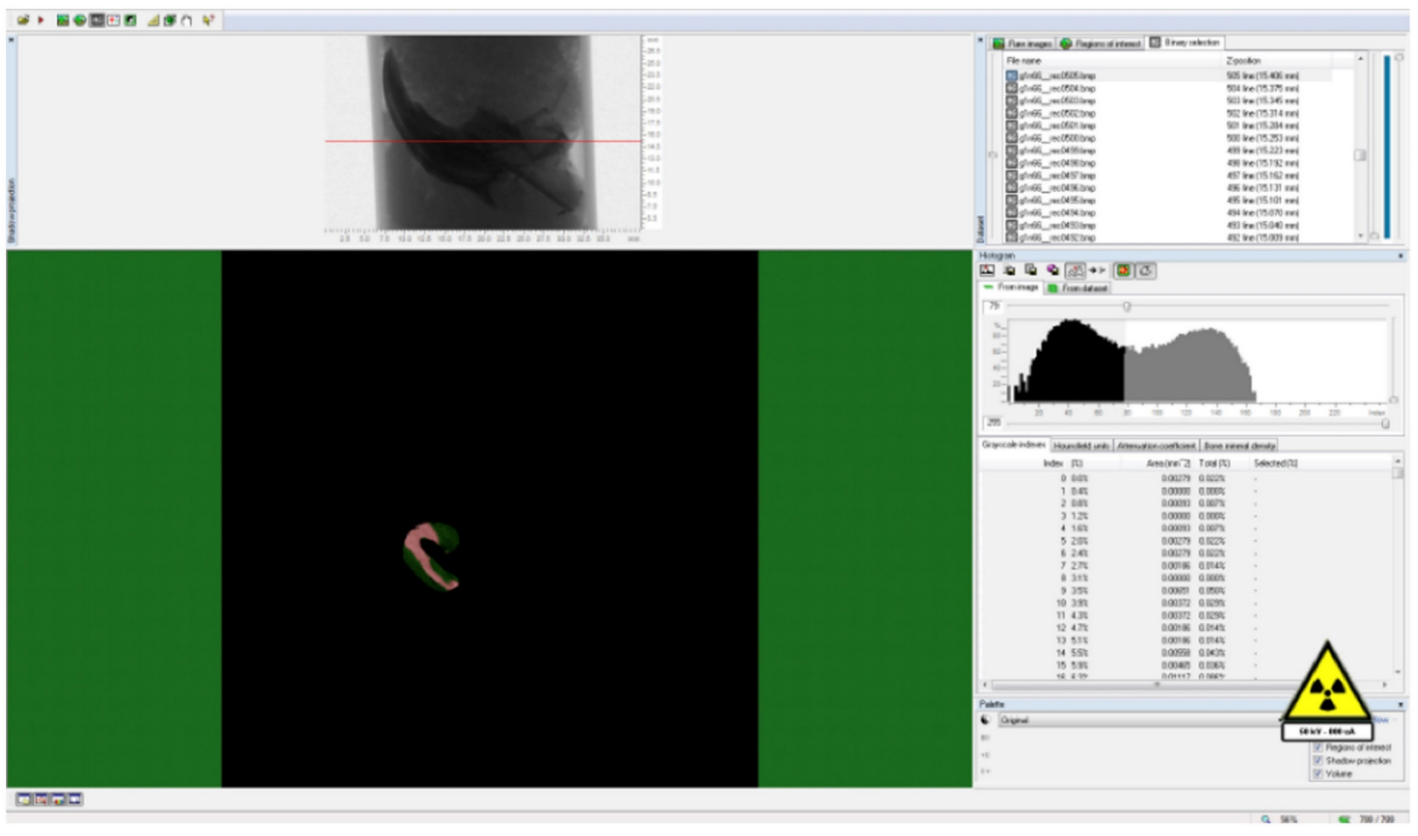
Determination of threshold value for the relevant field.

To delineate the boundaries of the region to be measured, a semi-automatic circular Region of Interest (ROI) was delineated in the horizontal plane on the image slices, as illustrated in Figure 3. A threshold value was ascertained within the histogram to differentiate between the voxels representing the sample under examination and those representing the surrounding air, as exemplified in Figure 4. Specifically, black voxels were assigned a value of 0, signifying the minimum intensity, while white voxels were assigned a value of 255, representing the maximum intensity.

Volumetric ratios were subsequently computed separately using the designated ROIs and the threshold data. To generate three-dimensional modeling images of the specimens, the data were transferred to the CTVol software (Version 2.3.2.0) (Bruker, Kontich, Belgium). A calcium hydroxyapatite (CaHA) calibration bar (phantom) was employed for the analysis, featuring calcium densities of 0.25 g/mm³ and 0.75 g/mm³. The CTAn software (Version 1.16.4.1+) (Bruker, Kontich, Belgium) was used to calculate measurements of bone mineral density (expressed in g/cm³) and bone volume (in cm³) within the specified region.

### Histopathological Analysis

After micro CT analysis, the mandibles of the rats were separated and fixed in 10% formalin solution (Fisher Scientific, Hampton, USA). Samples were decalcified in formic acid buffered with citrate for 48 hours and then embedded in paraffin. 5 µm-thick serial sections were taken from the region around the extraction socket in the longitudinal plane and stained with hematoxylin and eosin (HE) for histopathological examination. For each sample, three sections were examined for new bone formation, dead bone areas (without viable osteocytes), inflammatory cell infiltration, and epithelial cell regeneration under a light microscope at ×100 magnification (Olympus, Tokyo, Japan). The examination process was carried out by an experienced pathologist who was unaware of the study protocol.

### Statistical Analysis

The statistical analysis for this study was performed using the NCSS (Number Cruncher Statistical System) 2007 Statistical Software package program (NCSS, Utah, USA). Sample size was determined through a power analysis conducted using preliminary data, with the goal of achieving 80% power and an alpha level of 0.05. This calculation considered the effect size, variability, and the desired statistical power to ensure the reliability and validity of the results. The sample size determination process is detailed in the supplementary material provided (ARRIVE guidelines) 18.

In data analysis, descriptive statistics such as mean, standard deviation, median, and interquartile range were utilized to summarize the data. The distribution of variables was assessed using the Shapiro-Wilk normality test to determine if the data followed a normal distribution. For variables with normal distribution, one-way analysis of variance (ANOVA) was performed to compare the groups. Post-hoc analyses were conducted using the Tukey multiple comparison test to identify significant differences between groups. For variables that did not follow a normal distribution, non-parametric tests were used as appropriate. The details of the statistical methods and calculations, including any adjustments or corrections applied, are provided in the supplementary material.

## Results

Only three animals died due to excessive weight loss, so the experimental procedure was successfully completed by replacing the deceased ones. Biochemical, clinical, radiological, and histological findings are as follows.

### Biochemical Findings

**Figure 5:**
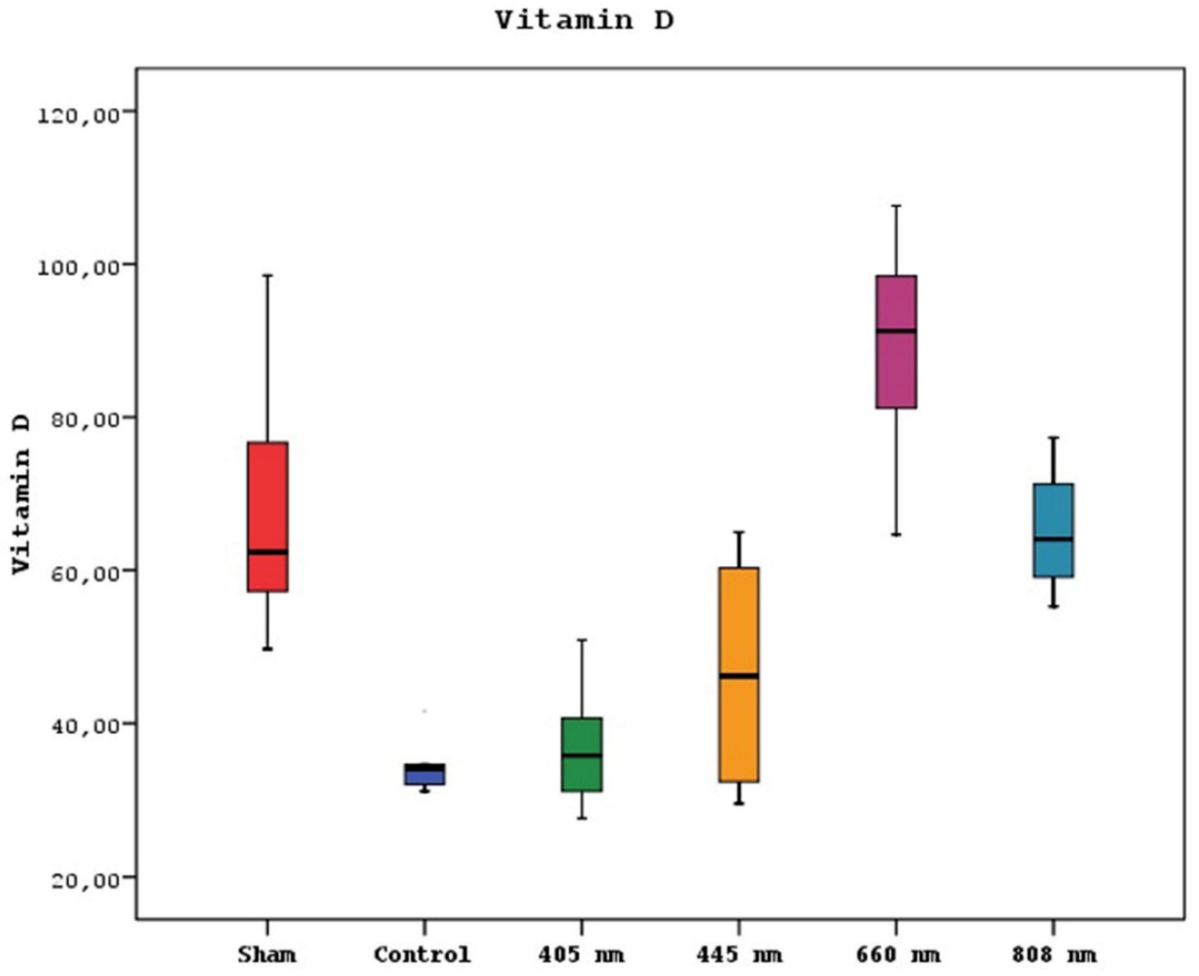
Mean serum Vitamin D levels in different study groups.

A statistically significant difference was observed between mean vitamin D levels in sham, control, 405 nm, 445 nm, 660 nm, and 808 nm groups (p=0.0001) (p=0.002). Serum vitamin D levels of the 660 nm group were significantly higher than sham, control, 405 nm, 445 nm, and 808 nm groups (p=0.006, p=0.0001). Mean vitamin D levels in the sham group were significantly higher than control, 405 nm, and 445 nm groups (p=0.008, p=0.0001). The level of the 808 nm group was considerably higher than the control, 405 nm, and 445 nm groups (p=0.025, p=0.0001). No statistically significant difference was observed between the other groups (p>0.05). The results of the serum vitamin D levels are given in Figure 5.

### Clinical Findings in Subjects

The subjects who underwent tooth extraction after repeat doses of zoledronic acid were examined regarding wound healing and divided into four groups (Table 3). Proper healing was seen in 20 subjects, while the hyperemic mucosal area was seen in 19 rats. Exposed bone area was seen in 7 subjects as only 2 remaining animals had abscess and suppuration.

A statistically significant difference was observed between the healing score distributions of the sham, control, 405 nm, 445 nm, 660 nm, and 808 nm groups (p=0.0001).

**Figure 6:**
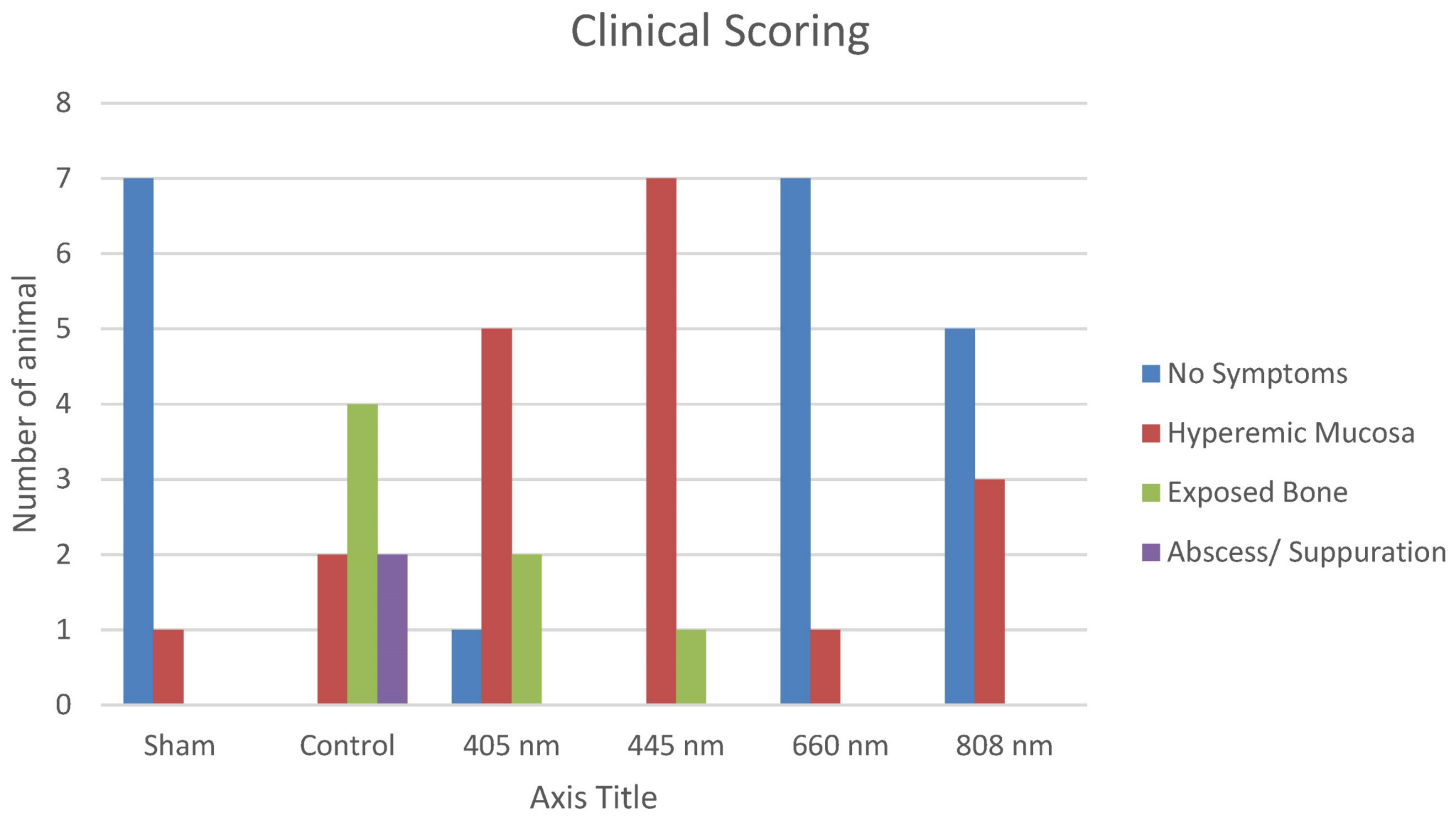
Clinical Scoring.

The normal healing presence distributions of sham, 660 nm, and 808 nm groups were higher than those of control, 405 nm, and 445 nm groups. Abscess distributions of the control group were higher than those of the sham, 405 nm, 445 nm, 660 nm, and 808 nm groups. The distribution of hyperemic tissue presence in 405 nm and 445 nm groups was higher than in sham, control, 660 nm, and 808 nm groups (Figure 6, Table 4,5,6).

### Radiological Findings

**Figure 7:**
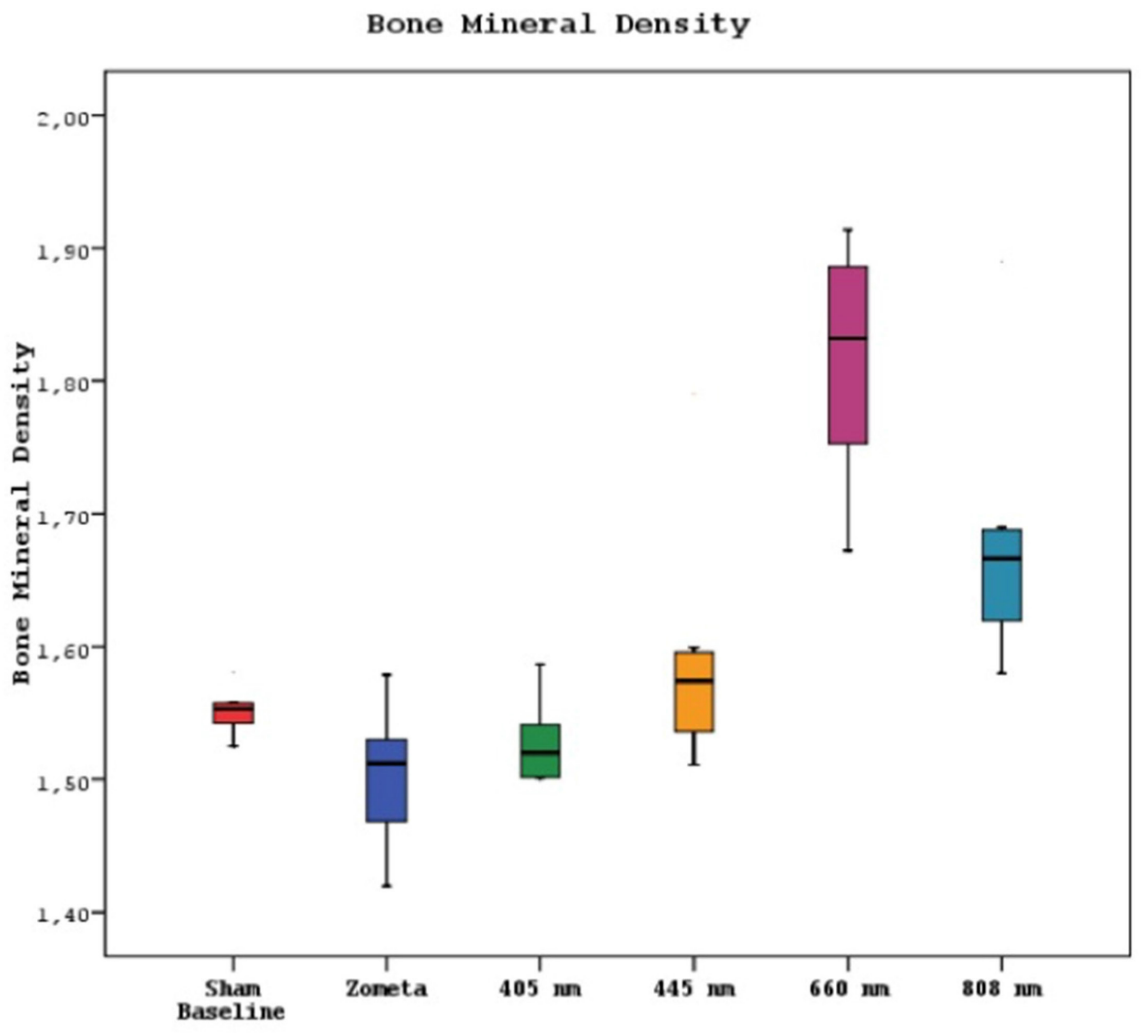
Mean bone mineral density levels in different study groups.

Micro CT examinations revealed a statistically significant difference between the bone mineral density means of sham, control, 405 nm, 445 nm, 660 nm, and 808 nm groups (p=0.0001). The bone mineral density mean of the 660 nm group was significantly higher than the other five groups (p=0.002, p=0.0001). The mean of the 808 nm group was substantially higher than the sham, control, and 405 nm groups (p=0.007, p=0.0001). No statistically significant difference was observed between the other groups (p>0.05) (Figure 7).

**Figure 8:**
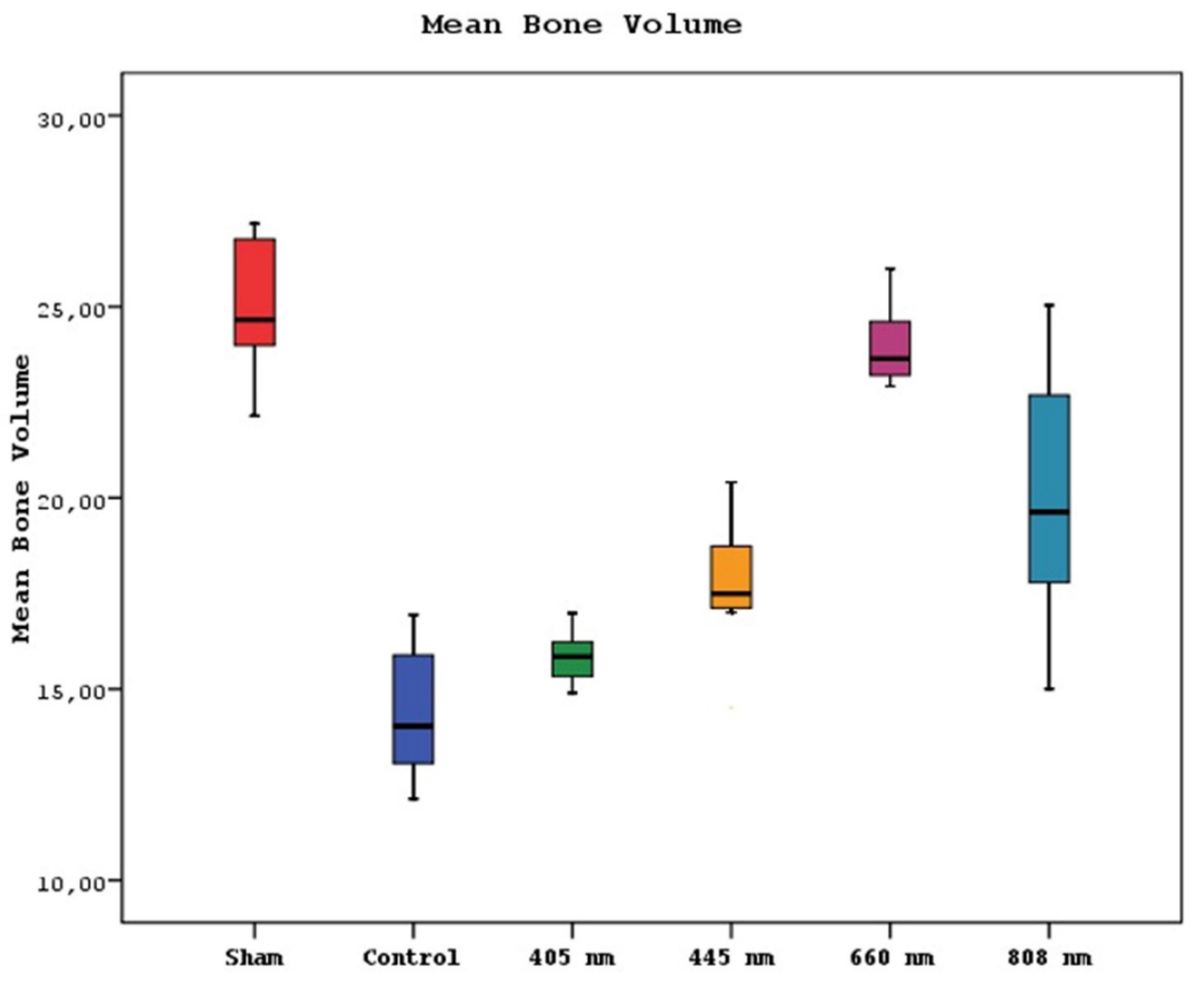
Mean bone volume levels in different study groups.

Furthermore, study groups exhibited significant differences in the mean volumes of bone(p=0.0001). The mean bone volume of the sham group was statistically significantly higher than the other groups except 660 nm(p=0.0001). The mean bone volumes of 660 nm and control groups were significantly higher than 405 nm, 445 nm, and 808 nm groups (p=0.002, p=0.0001). A significant difference in the mean bone volume of the 808 nm group was found, which presented a higher mean bone volume than the control and 405 nm groups (p=0.001, p=0.0001). The 445 nm group was also significantly higher than the control group according to mean bone volume (p=0.013). No statistically significant difference was observed between the other groups (p>0.05) (Figure 8).

### Histological Findings

**Figure 9:**
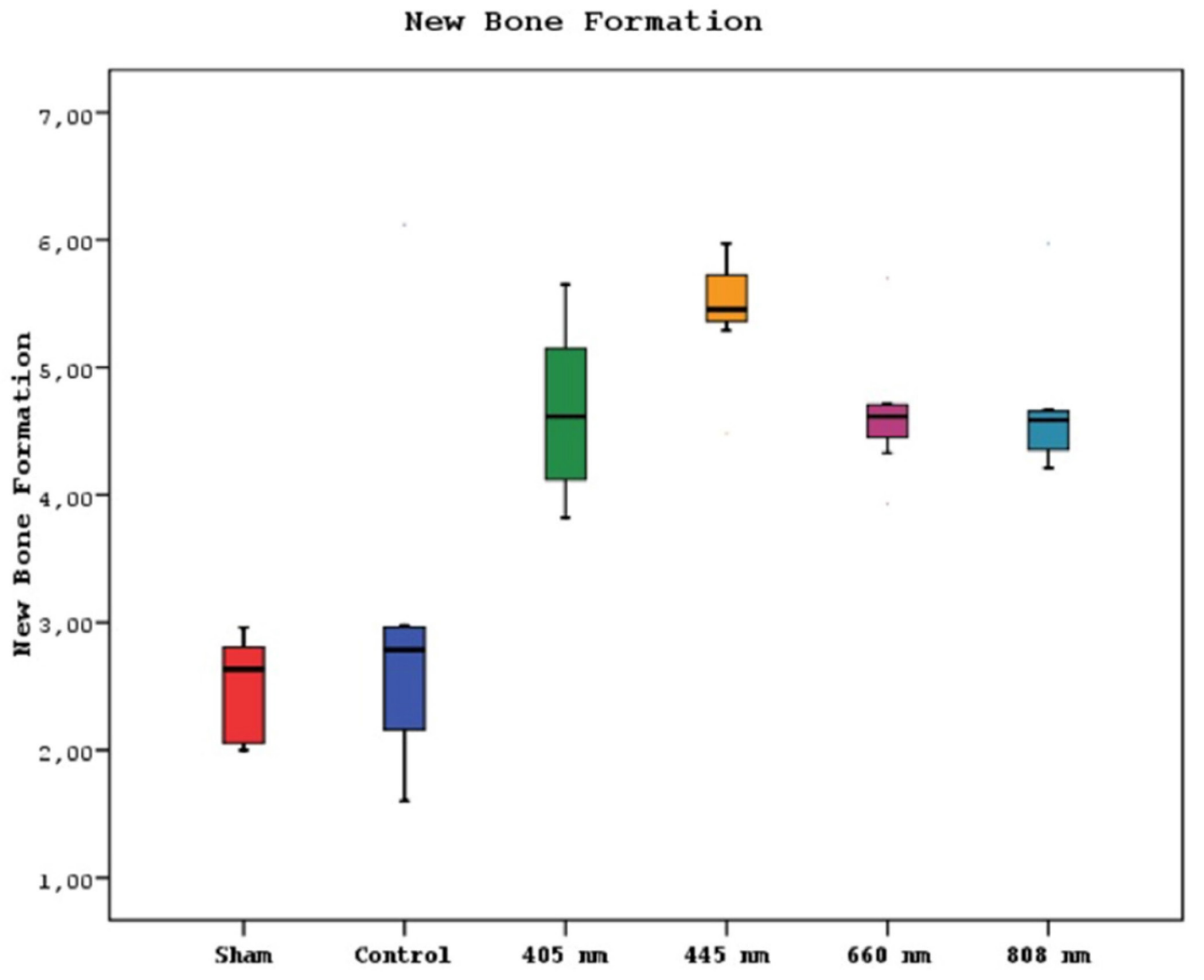
New bone formation levels in different study groups.

**Figure 10:**
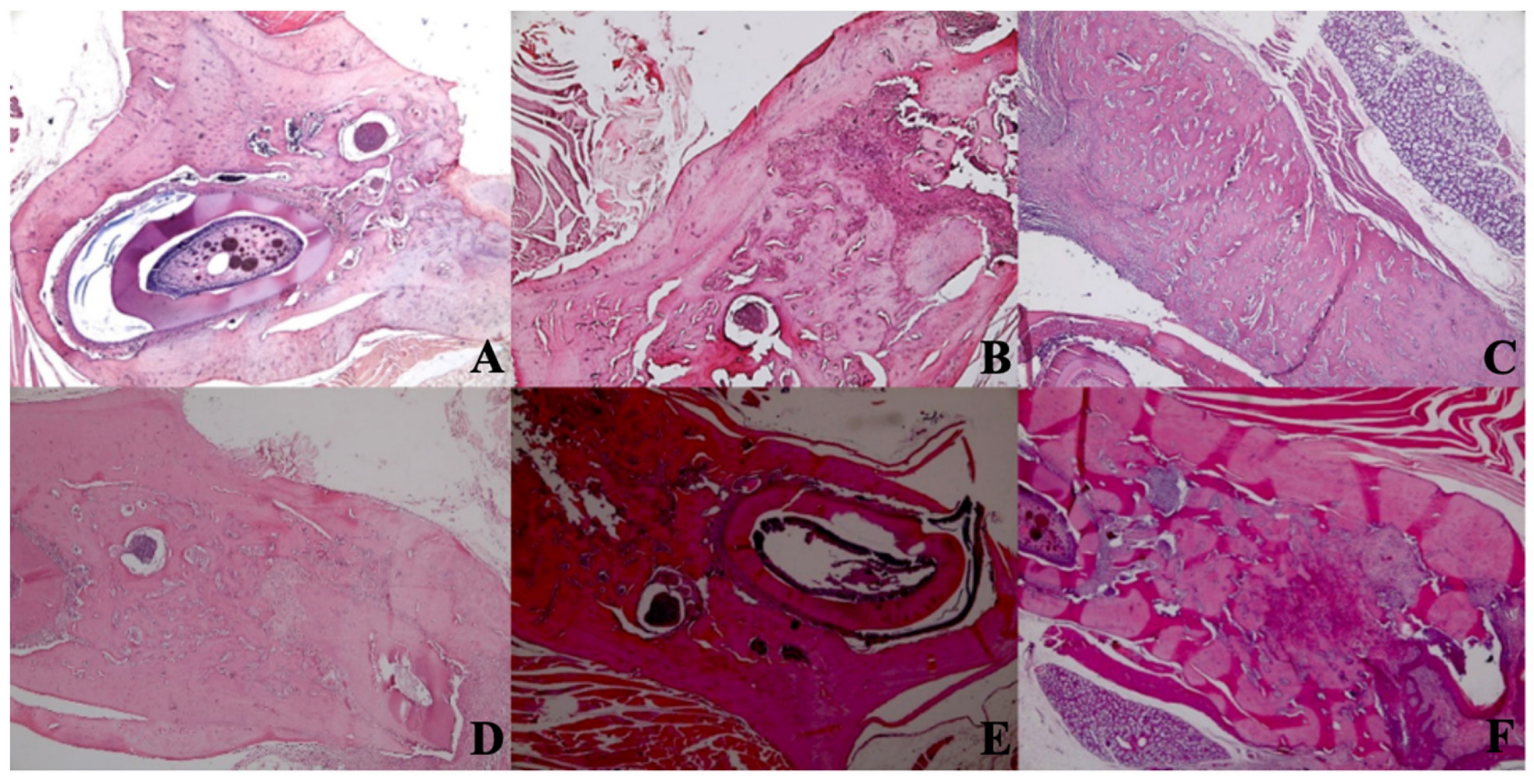
New bone formation. A In the SHAM group, there is a live bone in the form of bands in the defect area, on and around the tooth germ, areas of new bone formation in the environment, and dead bone areas in the near-surface areas. H&E, X40. B In the control group, there are dead bone fragments on the surface, live bone on both sides, and new bone formation areas around the granulation tissue in the middle section of the defect area. H&E, X40. C In the laser 405 nm, live bone in the defect area, lower margin, granulation tissue, and dead bone fragments at the left end and new bone formation in large areas are observed. H&E, X40. D In the laser 445 nm, dead bone, live bone, and new bone formation areas are observed in the defect area around the tooth, and a small amount of granulation tissue is observed. H&E; X40. E In the laser 660 nm, intense new bone formation is observed around the tooth, between the living bone areas. H&E; X40. F In the laser 808 nm, dense new bone formation and granulation tissue are seen between living bones. H&E; X40.

A statistically significant difference was observed between new bone formation, dead bone areas, epithelial cell regeneration, and inflammation cell rates of sham, control, 405 nm, 445 nm, 660 nm, and 808 nm groups (p=0.0001). The sham group's mean new bone formation rate was statistically significantly lower than the mean rates of the other groups except the control group(p=0.0001). In addition, the control group's rate was statistically significantly lower than the mean rates of the 405 nm, 445 nm, 660 nm, and 808 nm groups (p=0.001, p=0.0001). No statistically significant difference was observed between the other groups (p>0.05) (Figure 9,10).

**Figure 11:**
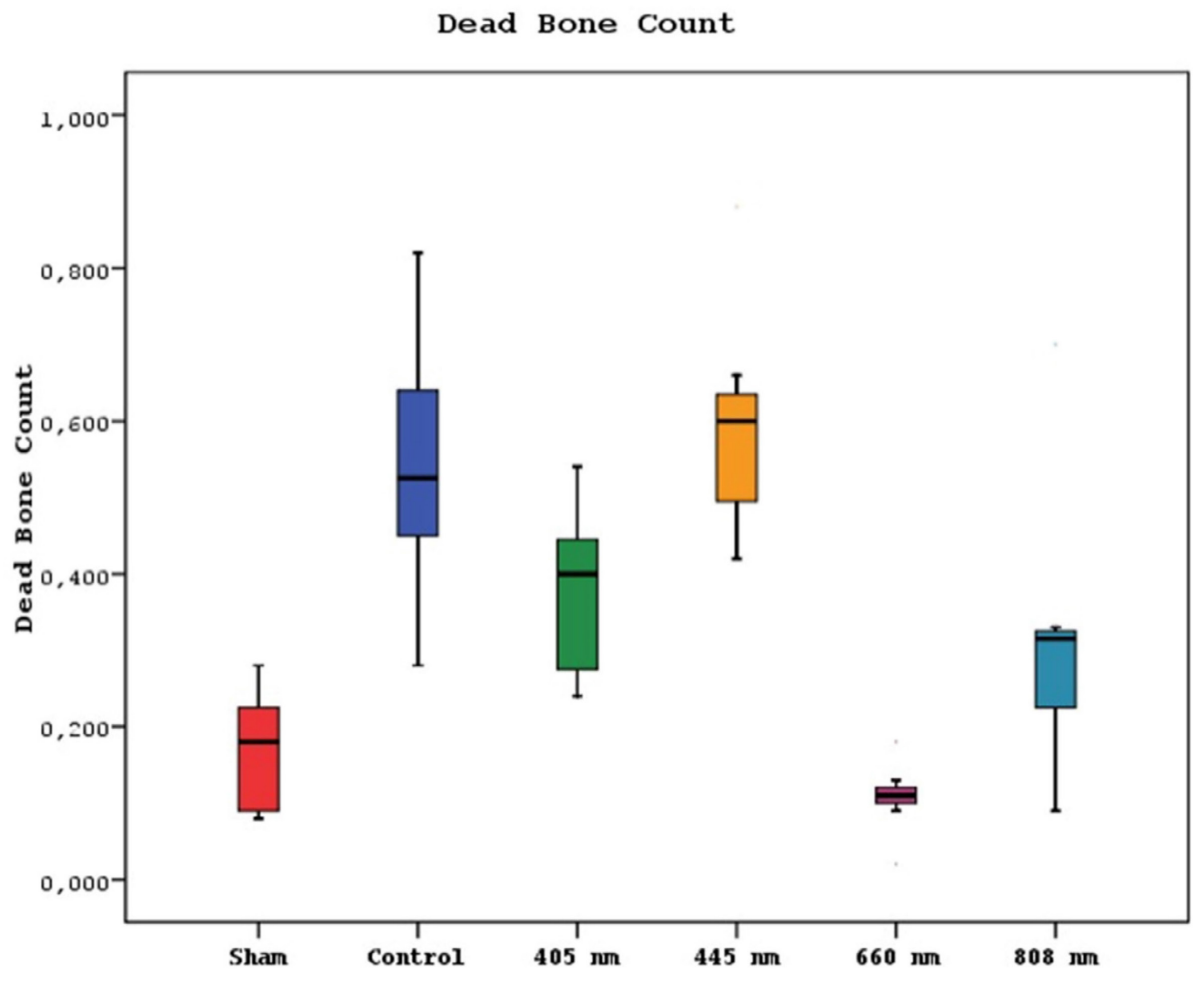
Mean dead bone counts in different study groups.

**Figure 12:**
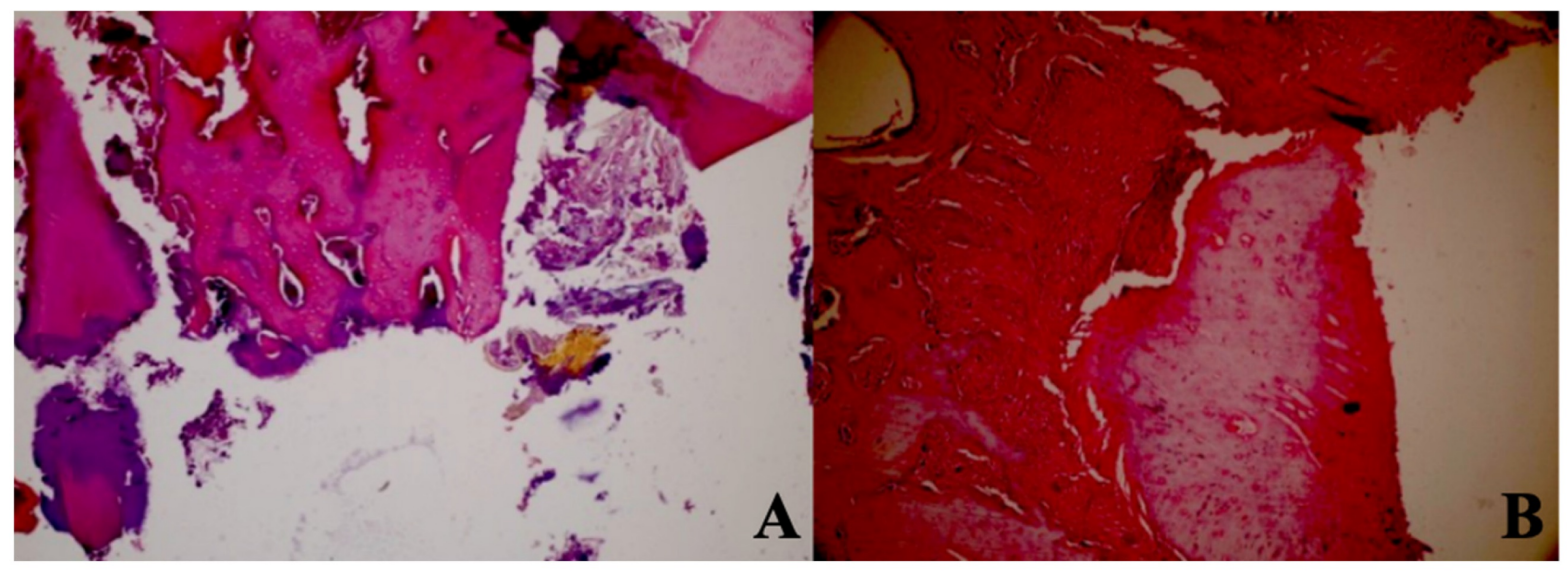
Dead bone count. A In the control group, granulation tissue, and minimal new bone formation were seen between large areas of dead bone. H&E; X40. B In the Laser 660 nm group, inflammation, and granulation tissue were observed around the dead bone fragments. H&E; x100.

Regarding the number of dead bones, the mean value of the 445 nm groups was statistically significantly higher than the mean values of the other groups except the control group (p=0.007, p=0.001). The control group's mean value was statistically significantly higher than the other groups, except for the 445 nm group (p=0.021, p=0.001). The rates of the 405 nm and 880 nm groups were significantly higher than the sham and 660 nm groups (p=0.002, p=0.001) (p=0,020, p=0,007). Additionally, while no statistically significant difference was observed between the other groups (p>0.05) (Figure 11,12).

**Figure 13:**
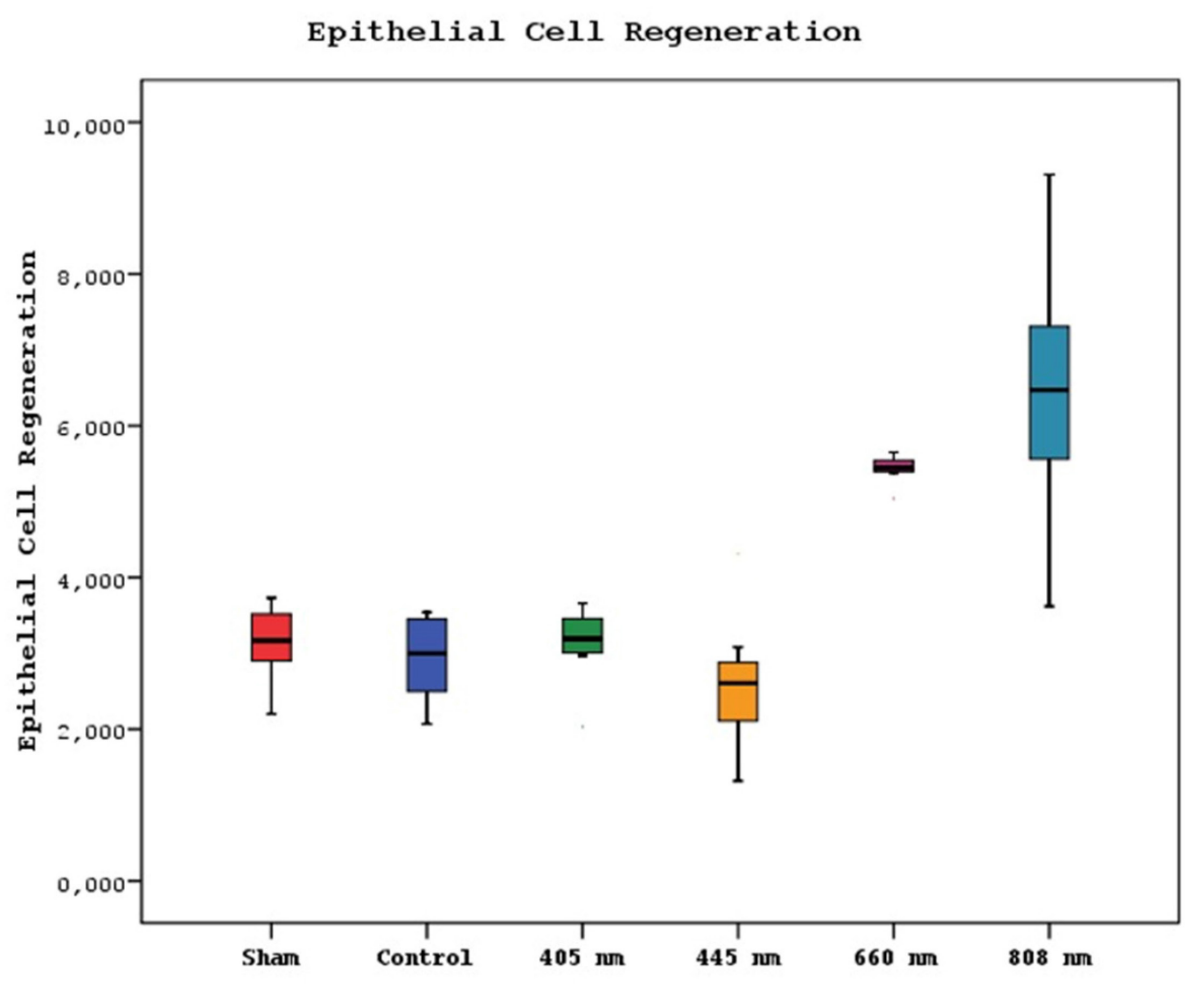
Mean epithelial cell regeneration values in different study groups.

**Figure 14:**
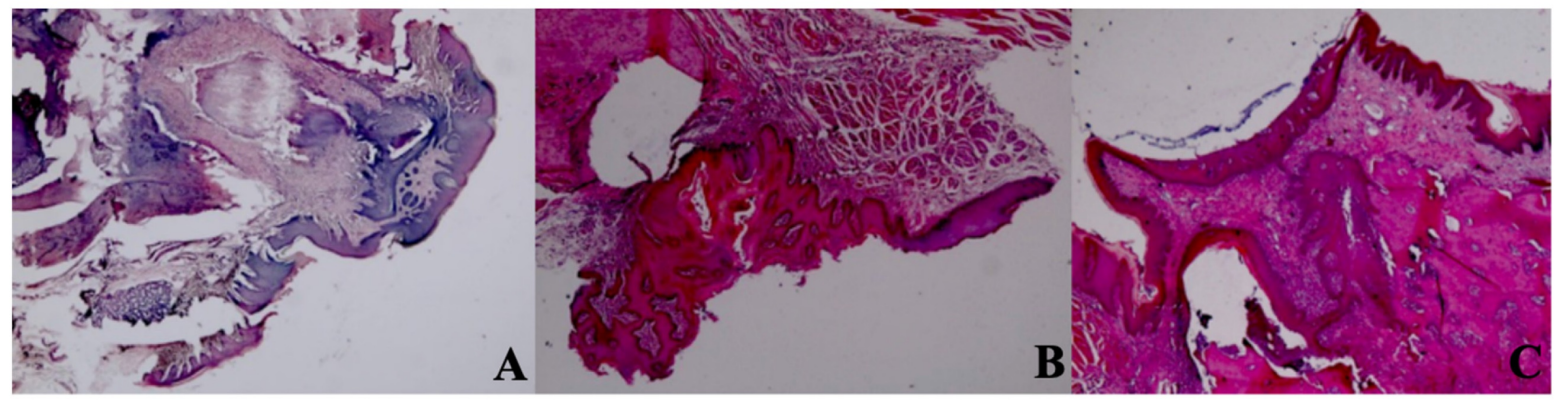
Epithelial cell regeneration. A In the control group, epithelial regeneration was observed on the wound surface. H&E; X40. B In the Laser 660 nm group, epithelial regeneration was observed on the wound surface. H&E; X40. C In the Laser 808 nm group, epithelial regeneration was observed that covers the defect area and surrounds the deep dead bone. H&E; X40.

Regarding the epithelial cell regeneration, the mean values in the 880 nm and 660 nm groups were significantly higher than in the sham, control, 405 nm, and 445 nm groups (p=0,001). No statistically significant difference was observed between the other groups (p>0.05) (Figure 13,14).

**Figure 15:**
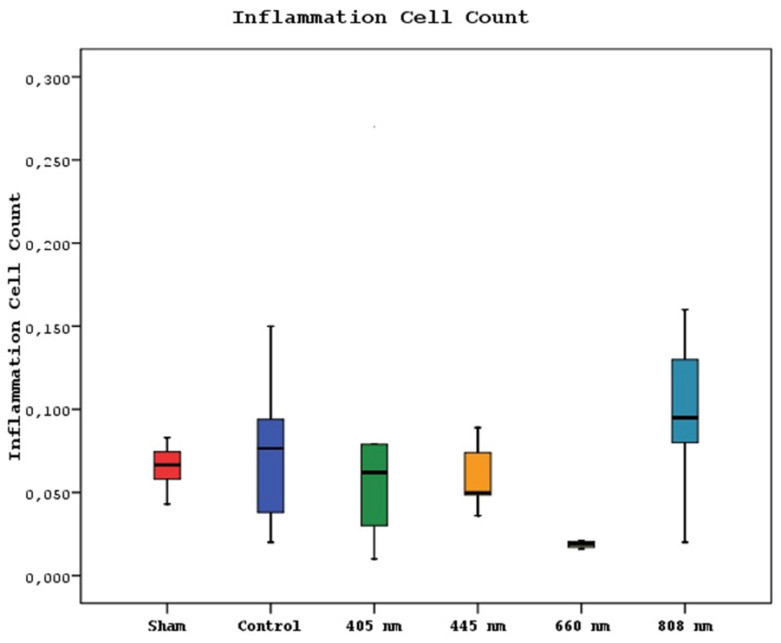
Mean inflammation cell counts in different study groups.

**Figure 16:**
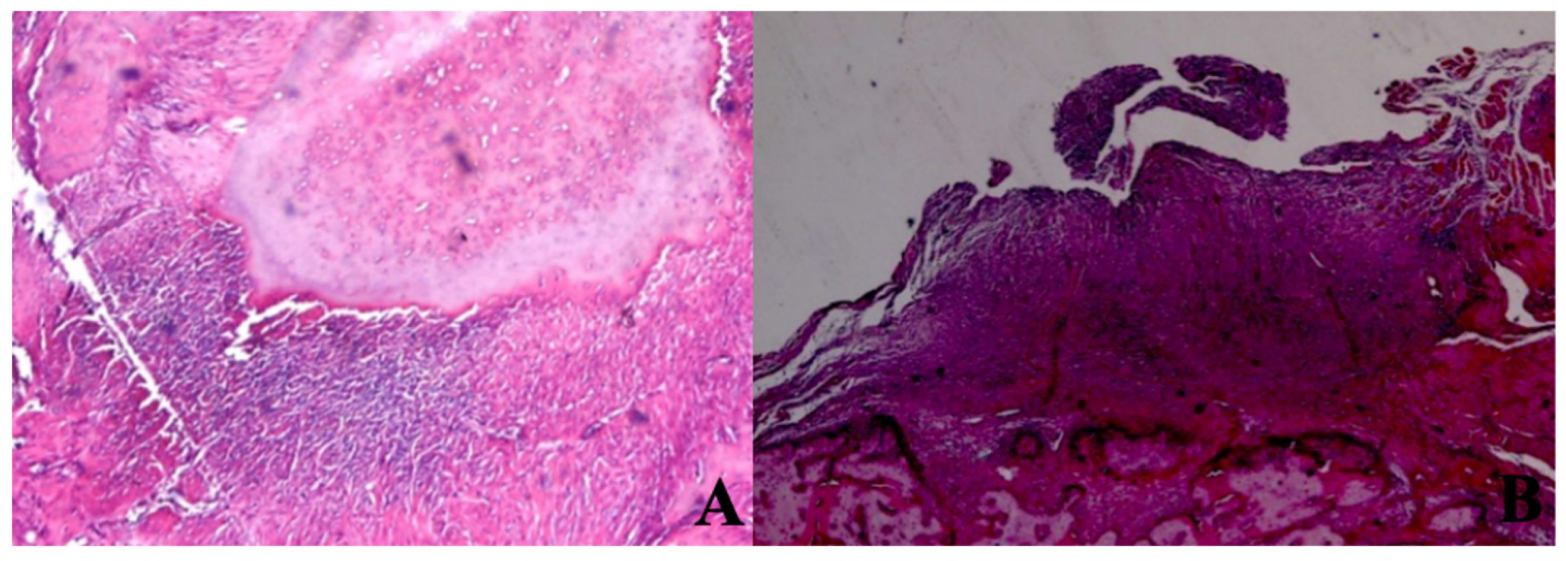
Inflammation Cell Count. A In the SHAM group, areas of inflammation were observed around dead bone fragments. H&E; x100. B In the 445 nm group, intense inflammation, and granulation tissue formation were observed around the new bone formation. H&E; x100.

The mean inflammation cell count of the 880 nm group was significantly higher than the mean values of the other groups except the control group (p=0.036, p=0.002). Furthermore, the mean value of the 660 nm group was found to be significantly lower than the mean values of the control, 405 nm, and 445 nm groups (p=0.001), while no statistically significant difference was observed between the other groups (p>0.05) (Figure 15,16).

## Discussion

The treatment of MRONJ remains a topic of ongoing debate within the medical world. Two main approaches are generally considered for treating MRONJ: interventional (surgical) and conservative strategies. The traditional approach aims to alleviate the patient's symptoms using various antibiotics, pain relievers, and mouthwashes. Conversely, surgical treatment aims to remove the necrotic area before it further enlarges.

While conservative treatment can successfully alleviate or eliminate symptoms in some patients, it may be insufficient in others. On the other hand, in some instances, surgical interventions can exacerbate the clinical course of the disease. This dilemma has prompted clinicians to explore alternative approaches that could assist in deciding between surgical or conservative treatment 22, 23.

Among these adjunctive therapies, specific treatments have gained prominence, including the application of bone marrow stem cells (BMSC), platelet-rich plasma (PRP) treatments, hyperbaric oxygen (HBO) therapy, and laser applications 24. It could be interesting to test photobiomodulation in combination with other adjuvant treatments such as ozonized gel and water, or probiotics in order to understand their mutual effect on MRONJ treatment 25-27.

Upon reviewing the existing literature, clinical and experimental studies investigating lasers' effectiveness in treating medication-related osteonecrosis of the jaw (MRONJ) have been identified. Published clinical studies have reported successful outcomes in supporting surgical and medical treatment when lasers were retrospectively used 28. In a retrospective study by Vescovi *et al.*, combined medical treatment and photobiomodulation resulted in higher success rates than patients receiving only medical treatment. Additionally, combining traditional surgery and photobiomodulation yielded better results than conventional surgery alone 29. Furthermore, a meta-analysis conducted by Momesso *et al.* reported that laser surgery achieved a success rate of 90% in MRONJ patients. In comparison, the combination of classical surgical treatment and photobiomodulation achieved a 73.6% success rate. Solely relying on surgical treatment resulted in a success rate of 69%, while patients receiving only medical treatment had a notably low success rate of 18% 30.

Moreover, research has explored the effects of lasers on osteoblast cell proliferation and migration, vascularization, and inflammatory cells and has consistently yielded positive and significant results in various *in vivo* and *in vitro* settings 31-34. However, comparative studies examining the effects of lasers on MRONJ treatment across different wavelengths are outside our current knowledge. With this background in mind, our study aims to investigate the effects of lasers at four different wavelengths on both hard and soft tissues in treating MRONJ. We will conduct histopathological, radiological, biochemical, and clinical assessments to evaluate the impact of lasers on MRONJ treatment comprehensively.

In our study, it is possible to assert that the clinical assessment conducted on animals before their sacrifice confirms the efficacy of our MRONJ induction protocol. Specifically, following the protocol established in a study by Zandi and colleagues 19, animals in the CONTROL group received intraperitoneal (IP) zoledronic acid for four weeks, followed by tooth extraction. Subsequently, these animals were observed for eight weeks, during which MRONJ typically develops. After 8 weeks, clinical observations in the CONTROL group revealed that 4 out of 8 animals exhibited exposed bone at the extraction site, 2 showed abscess formation during palpation, and 2 displayed areas of disrupted hyperemic mucosa around the extraction socket.

The observations in the SHAM group, where 7 animals exhibited routine healing, further validate the effectiveness of our protocol. Additionally, the substantial disparity in the amount of necrotic bone observed between the CONTROL and SHAM groups, along with significant differences in serum vitamin D levels and average bone volume, suggests the successful induction of MRONJ in the animals. Moreover, while examining the clinical scoring values, it becomes evident that groups utilizing lasers with wavelengths of 660nm and 808nm exhibited results similar to the SHAM group. Contrarily, an analysis of clinical scores suggests that the 405nm and 445nm lasers may not be sufficiently effective for clinical MRONJ treatment. In summary, our study results support the effectiveness of the MRONJ induction protocol, with apparent differences between the CONTROL and SHAM groups. Furthermore, specific laser wavelengths appear more promising in treating MRONJ based on their clinical scoring results. However, the 405nm and 445nm lasers may require further investigation or alternative applications in MRONJ treatment due to their less favorable clinical outcomes.

Based on the results of our study, it is clear that lasers with wavelengths of 660nm and 808nm substantially impacted increasing serum vitamin D levels in rats afflicted with MRONJ. Notably, the 660nm laser surpassed vitamin D levels observed in the SHAM group. However, the precise mechanism underlying the influence of lasers on serum vitamin D levels remains a topic necessitating additional investigation. The observation that lasers with specific wavelengths, such as 660nm and 808nm, might impact vitamin D levels requires further exploration as the visible and near-infrared wavelengths used in this study do not directly influence vitamin D synthesis, which is typically stimulated by ultraviolet light 35.

In the micro CT analysis of bone density, it is observed that the 660nm group exhibits the highest values. The 808nm wavelength also significantly surpasses the bone density of the SHAM, CONTROL, and 405nm groups. Furthermore, the measurements of average bone volume in the 660nm group are nearly similar to those of the SHAM group. These findings are consistent with the results of clinical scoring and vitamin D levels. This alignment between the micro CT results, clinical scoring, and vitamin D measurements suggests a strong correlation between the laser wavelengths and their effects on bone density and overall outcomes in MRONJ treatment.

In our study, when examining histological sections, we observe significantly higher values for new bone formation in all laser groups compared to the CONTROL and SHAM groups. This finding aligns with existing literature that indicates lasers can enhance osteoblast proliferation and differentiation in cell culture studies 36-39. Moreover, experimental studies on animal models have reported that laser applications stimulate new bone formation 40, 41.

Based on our results, the 445nm wavelength laser had the most pronounced effect on new bone formation, indicating a positive impact among the laser groups. However, the 445nm group also exhibited the highest values of dead bone. This suggests that while the 445nm wavelength laser enhances new bone formation, its impact on dead bone quantity should be considered when evaluating laser efficacy for MRONJ treatment. Future research should focus on balancing new bone formation and dead bone quantity across different laser wavelengths.

The 660nm wavelength laser group showed the lowest amount of dead bone, even lower than the SHAM group, and the 808nm group also exhibited lower results compared to the CONTROL group. Although there are no studies directly explaining the reduction of dead bone with the 660nm laser, the survey by Tani *et al.* (2018) highlights that only the 635nm laser increased vinculin protein production in osteoblasts, which is crucial for cell-matrix and cell-cell adhesion. This increase in vinculin activity likely contributes to the reduced amount of dead bone observed in the 660nm laser group 42, 43.

Considering both dead bone and new bone formation, the use of 660nm and 808nm wavelengths in MRONJ treatment may yield favorable clinical outcomes. The 660nm wavelength laser group had the least inflammatory cell counts, which supports our study's findings on new bone formation, micro CT, and clinical scoring. This observation is consistent with previous research on lasers' effects on inflammation. For instance, Honmura *et al.* (1992) demonstrated the anti-inflammatory efficacy of diode lasers with a 780nm wavelength, and Huang *et al.* (2012) found that photobiomodulation with a 920nm laser suppressed inflammatory markers such as iNOS, TNF-α, and IL-1 44, 45.

Choi *et al.* (2012) showed that 635nm lasers suppress inflammation by reducing cytokine release, while Lopes-Martins *et al.* (2011) reported that a 650nm laser significantly decreased leukocyte and neutrophil counts. These findings align with our observation that the 660nm laser group had the lowest inflammatory cell count 46, 47.

Regarding epithelial regeneration, studies generally highlight the effectiveness of 660nm and 808nm lasers. Our study corroborates Amaroli *et al.* (2022) and Topaloglu *et al.* (2023), showing a limited increase in epithelial regeneration with the 660nm laser and more pronounced effects with the 808nm laser 7, 48. Conversely, the 445nm laser had a detrimental impact on epithelial regeneration, suggesting a negative influence on this regenerative process.

One limitation of our study is the relatively small number of experimental animals due to ethical considerations. Additionally, we used the same dosages across groups, limiting our ability to explore the effects of different doses. Future studies should investigate the impact of varying doses, particularly with the 660nm and 808nm wavelengths, to provide more comprehensive insights.

In conclusion, both the 660nm and 808nm wavelengths show positive effects in MRONJ treatment. Combining these wavelengths could be beneficial, as the 660nm wavelength appears more effective in bone tissue while the 808nm wavelength excels in soft tissue. However, further research is needed to understand the mechanisms through which lasers affect vitamin D levels and their role in MRONJ development and progression. Future studies should explore these aspects and the potential for combined laser therapies in MRONJ treatment.

## Supplementary Material

Supplementary information.

## Figures and Tables

**Table 1:** Study groups, wavelengths, and animal numbers.

Groups	Central Wavelength	Number Of Test Subjects (n)
Sham	n/a	8
Control	n/a	8
Laser 1	405nm	8
Laser 2	445nm	8
Laser 3	660nm	8
Laser 4	808nm	8

**Table 2:** Laser Technical Parameters

	Laser 1	Laser 2	Laser 3	Laser 4
Central Wavelength	405nm	445nm	660nm	808nm
Wavelength Accuracy	±2nm	±2nm	±2nm	±4nm
Laser Structure	InGaN	InGaN	AlGaInP	GaAlAs
Type of Energy Source	Diode	Diode	Diode	Diode
Beam Delivery Method	Hand Probe	Hand Probe	Hand Probe	Hand Probe
Beam Profile	Gaussian	Gaussian	Gaussian	Gaussian
Average Beam Power	120mw	120mw	120mw	120mw
Aperture	5mm x 5mm	5mm x 5mm	5mm x 5mm	5mm x 5mm
Operation Mode	Continuous Wave	Continuous Wave	Continuous Wave	Continuous Wave
Beam Shape	Square	Square	Square	Square
Illumination in the Diaphragm	480mw/cm^2^	480mw/cm^2^	480mw/cm^2^	480mw/cm^2^
Area of Application	0.25cm^2^	0.25cm^2^	0.25cm^2^	0.25cm^2^
Laser irradiation Dose	6Joule/cm^2^	6Joule/cm^2^	6Joule/cm^2^	6Joule/cm^2^
Irradiation Period (Single Session)	16 Second	16 Second	16 Second	16 Second
Energy Delivered in one Session	1.5 Joule	1.5 Joule	1.5 Joule	1.5 Joule
Total Number of Sessions	8	8	8	8
Session Frequency	Week / 2	Week / 2	Week / 2	Week / 2
Total Energy Intensity	48Joule/cm^2^	48Joule/cm^2^	48 Joule/cm^2^	48Joule/cm^2^
Total Application Time	128 Second	128 Second	128 Second	128 Second
Total Net Energy Delivered	12 Joule	12 Joule	12 Joule	12 Joule

**Table 3:** Clinical findings.

	No Symptoms	Hyperemic Mucosa	Exposed Bone	Abscess/Suppuration
Sham (1-8)	7	1	0	0
Control (9-16)	0	2	4	2
405nm (17-24)	1	5	2	0
445nm (25-32)	0	7	1	0
660nm (33-40)	7	1	0	0
808nm (41-48)	5	3	0	0
Total	20	19	7	2

**Table 4:** Statistical evaluation of clinical findings (Chi-Square test)

Clinical Scoring	No Symptoms		Hyperemic Mucosa		Exposed Bone		Abscess/ Suppuration		p
**Sham**	7	87.50%	1	12.50%	0	0.00%	0	0.00%	0.0001
**Control**	0	0.00%	2	25.00%	4	50.00%	2	25.00%	
**405 nm**	1	12.50%	5	62.50%	2	25.00%	0	0.00%	
**445 nm**	0	0.00%	7	87.50%	1	12.50%	0	0.00%	
**660 nm**	7	87.50%	1	12.50%	0	0.00%	0	0.00%	
**808 nm**	5	62.50%	3	37.50%	0	0.00%	0	0.00%	

**Table 5:** One-Way Analysis of Variance

	Vitamin D	New Bone Formation	Bone Mineral Density	Mean Bone Volume	Epithelial Cell Regeneration	Inflammation Cell Count	Dead Bone Count
Sham	67.58±15,75	2.49±0.40	1.55±0.02	25.02±1.77	3.139±0.492	0.066±0.013	0.169±0.077
Control	34.29±3,25	2.94±1.39	1.50±0.05	14.37±1.81	2.939±0.542	0.073±0.042	0.541±0.167
405 nm	36.72±7,58	4.65±0.68	1.53±0.03	15.84±0.66	3.125±0.503	0.078±0.082	0.378±0.105
445 nm	46.52±14,22	5.44±0.45	1.59±0.09	17.70±1.73	2.603±0.873	0.059±0.019	0.595±0.144
660 nm	89.24±14,43	4.65±0.50	1.82±0.09	23.98±1.05	5.431±0.183	0.019±0.006	0.108±0.044
808 nm	65.16±7,74	4.67±0.55	1.68±0.09	20.03±3.29	6.451±1.757	0.099±0.044	0.315±0.175
p*	0.0001	0.0001	0.0001	0.0001	0.0001	0.001	0.0001

**Table 6:** Tukey Multiple Comparison Test

Tukey Multiple Comparison Test	Vitamin D	Bone Mineral Density	Mean Bone Volume	New Bone Formation	Dead Bone Count	Epithelial Cell Regeneration	Inflammation Cell Count
Sham / Control	**0.0001**	0.697	**0.0001**	0.831	**0.001**	0.372	0.563
Sham / 405 nm	**0.0001**	0.976	**0.0001**	**0.0001**	**0.002**	0.875	0.753
Sham / 445 nm	**0.008**	0.873	**0.0001**	**0.0001**	**0.001**	0.074	0.293
Sham / 660 nm	**0.006**	**0.0001**	0.882	**0.0001**	0.244	**0.001**	**0.001**
Sham / 808 nm	0.998	**0.007**	**0.0001**	**0.0001**	**0.02**	**0.001**	**0.036**
Control / 405 nm	0.998	0.979	0.644	**0.0001**	**0.031**	0.431	0.494
Control / 445 nm	0.289	0.129	**0.013**	**0.0001**	0.43	0.345	0.713
Control / 660 nm	**0.0001**	**0.0001**	**0.0001**	**0.001**	**0.001**	**0.001**	**0.003**
Control / 808 nm	**0.0001**	**0.0001**	**0.0001**	**0.0001**	**0.021**	**0.001**	0.188
405 nm / 445 nm	0.531	0.445	0.382	0.297	**0.007**	0.074	0.916
405 nm / 660 nm	**0.0001**	**0.0001**	**0.0001**	0.999	**0.001**	**0.001**	**0.023**
405 nm / 808 nm	**0.0001**	**0.001**	**0.001**	0.999	0.207	**0.001**	0.103
445 nm / 660 nm	**0.0001**	**0.0001**	**0.0001**	0.287	**0.001**	**0.001**	**0.001**
445 nm / 808 nm	**0.025**	0.119	0.164	0.319	**0.009**	**0.001**	**0.021**
660 nm / 808 nm	**0.002**	**0.002**	**0.002**	0.999	**0.007**	0.093	**0.002**
