# Supplementary material for: Comparison of the Effects of Four Laser Wavelengths on Medication-Related Osteonecrosis of the Jaw (MRONJ) in a Murine Model: An In Vivo Photobiomodulation Study
Source: Int J Med Sci. 2024 Nov 11;21(15):2959–73. doi: 10.7150/ijms.93224 (PMC11610323; doi:10.7150/ijms.93224)

Raw Data and Images of 93224n2

	subject	sample	RANKLE	Vit D	New bone formation	reverse scoring	Bone Mineral Density	average bone volume	Epithelial Cell Regeneration	Inflammation Cell Count	Dead Bone Count
sham baseline	1	2	7,832	58,947	2,00	1	1,551	23,976	2,750	0,055	0,21
sham baseline	2	3	4,554	55,566	2,75	1	1,558	26,416	3,460	0,083	0,28
sham baseline	3	4	4,172	61,593	2,81	1	1,525	27,175	3,130	0,061	0,23
sham baseline	4	5	9,002	98,490	2,06	0	1,534	27,111	3,580	0,071	0,08
sham baseline	5	7	5,077	73,353	2,96	1	1,557	24,310	3,210	0,062	0,10
sham baseline	6	13	9,302	79,968	2,52	1	1,555	24,009	3,050	0,076	0,22
sham baseline	7	14	8,197	49,686	2,80	1	1,581	22,150	3,730	0,073	0,15
sham baseline	8	15	8,965	63,063	2,05	1	1,551	25,009	2,200	0,043	0,08
zometa	9	68	8,061	34,298	2,94	2	1,481	12,342	3,050	0,088	0,53
zometa	10	69	8,927	31,160	2,84	3	1,456	13,768	2,070	0,150	0,82
zometa	11	70	9,649	32,490	2,98	2	1,538	16,890	3,380	0,046	0,44
zometa	12	71	6,297	41,600	2,56	1	1,522	14,053	2,430	0,100	0,28
zometa	13	72	8,002	31,610	1,60	2	1,514	16,938	3,520	0,073	0,46

zometa	14	73	3,930	33,950	1,76	2	1,579	12,124	3,540	0,020	0,56
zometa	15	74	9,763	34,690	2,73	3	1,510	14,879	2,570	0,030	0,72
zometa	16	75	9,844	34,550	6,12	1	1,420	14,002	2,950	0,080	0,52
405nm	25	82	8,859	43,941	3,96	1	1,501	16,234	3,550	0,010	0,29
405nm	26	83	5,346	32,046	5,61	2	1,544	15,765	2,970	0,270	0,38
405nm	27	84	5,551	27,636	4,28	1	1,502	16,982	3,360	0,040	0,42
405nm	28	85	5,064	30,282	3,82	1	1,536	15,321	2,030	0,020	0,26
405nm	29	86	10,646	50,862	5,65	0	1,538	14,897	3,660	0,050	0,24
405nm	30	87	10,921	34,839	4,65	1	1,501	15,345	3,120	0,079	0,45
405nm	31	233	7,742	36,754	4,69	2	1,587	15,923	3,260	0,074	0,54
405nm	32	234	7,219	37,373	4,58	1	1,504	16,234	3,050	0,079	0,44
445nm	41	95	2,586	44,394	5,62	2	1,579	19,234	3,080	0,036	0,56
445nm	42	96	3,499	59,535	5,83	1	1,599	20,410	1,940	0,050	0,61
445nm	43	97	6,776	64,974	5,46	1	1,569	17,354	1,320	0,050	0,42
445nm	44	98	4,528	47,902	5,97	1	1,532	14,521	2,680	0,060	0,66
445nm	45	99	4,146	29,547	5,29	1	1,511	17,003	2,280	0,088	0,43

445nm	46	100	4,172	33,520	5,45	1	1,790	17,638	2,600	0,049	0,60
445nm	47	101	3,664	61,005	4,48	1	1,592	18,234	4,310	0,089	0,88
445nm	48	102	3,283	31,311	5,43	1	1,540	17,239	2,610	0,048	0,60
660nm	57	117	7,153	92,316	5,70	0	1,830	24,235	5,040	0,016	0,02
660nm	58	118	6,414	104,517	4,60	0	1,898	23,345	5,410	0,018	0,11
660nm	59	119	6,414	91,728	4,33	0	1,672	23,845	5,650	0,010	0,18
660nm	60	120	5,205	107,604	3,93	1	1,875	22,923	5,600	0,030	0,13
660nm	61	121	4,783	64,680	4,70	0	1,786	23,435	5,460	0,020	0,09
660nm	62	122	7,192	72,912	4,63	0	1,914	25,991	5,430	0,021	0,11
660nm	63	237	6,182	90,733	4,71	0	1,834	24,993	5,480	0,018	0,11
660nm	64	238	6,194	89,457	4,57	0	1,720	23,078	5,380	0,020	0,11
808nm	73	125	9,337	61,152	4,31	1	1,580	22,941	3,620	0,020	0,70
808nm	74	126	8,265	55,254	4,54	0	1,619	25,042	9,310	0,090	0,23
808nm	75	127	7,296	57,036	4,64	0	1,620	17,234	6,450	0,090	0,32
808nm	76	128	7,075	77,322	5,97	0	1,690	15,002	4,700	0,070	0,09
808nm	77	129	4,860	68,502	4,21	1	1,650	19,234	7,970	0,150	0,33

808nm	78	130	3,220	73,941	4,40	0	1,682	18,346	6,650	0,160	0,22
808nm	79	131	7,871	63,543	4,63	1	1,686	22,435	6,420	0,110	0,31
808nm	80	245	6,899	64,532	4,67	0	1,890	20,032	6,490	0,100	0,32

0 , no symptoms
1, hyperemic tissue
2, Presence of exposed bone
3, presence of abscess

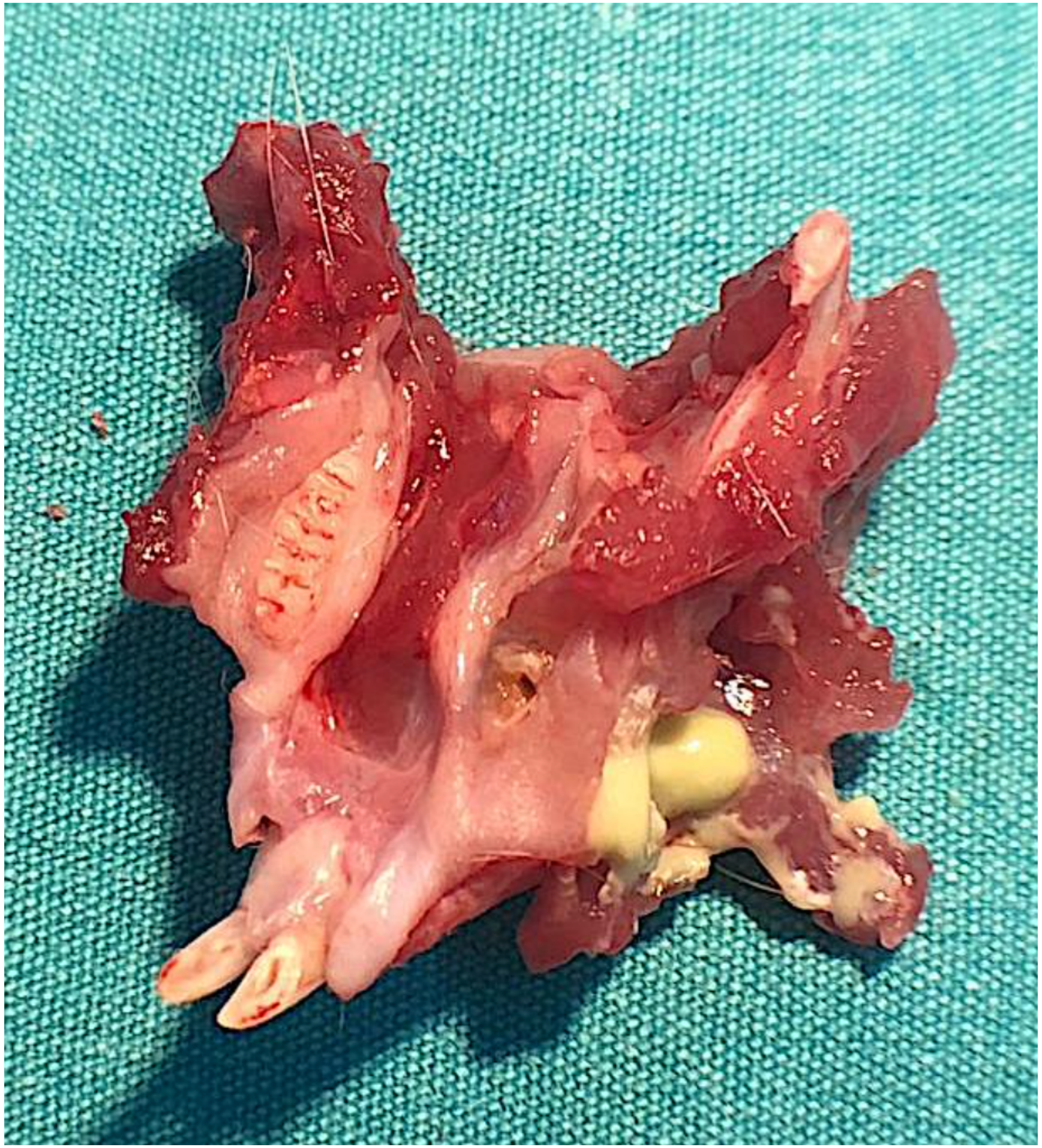

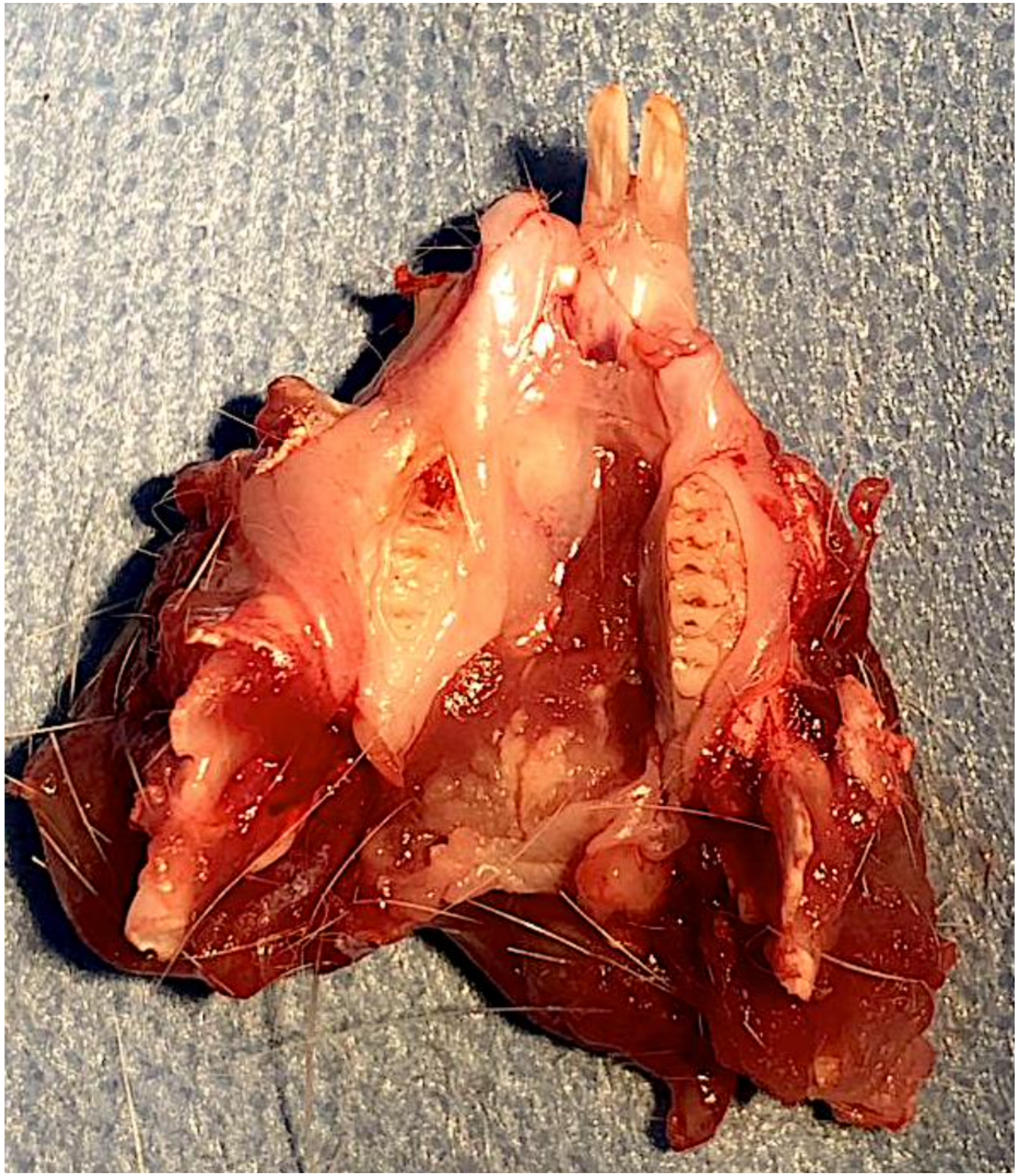

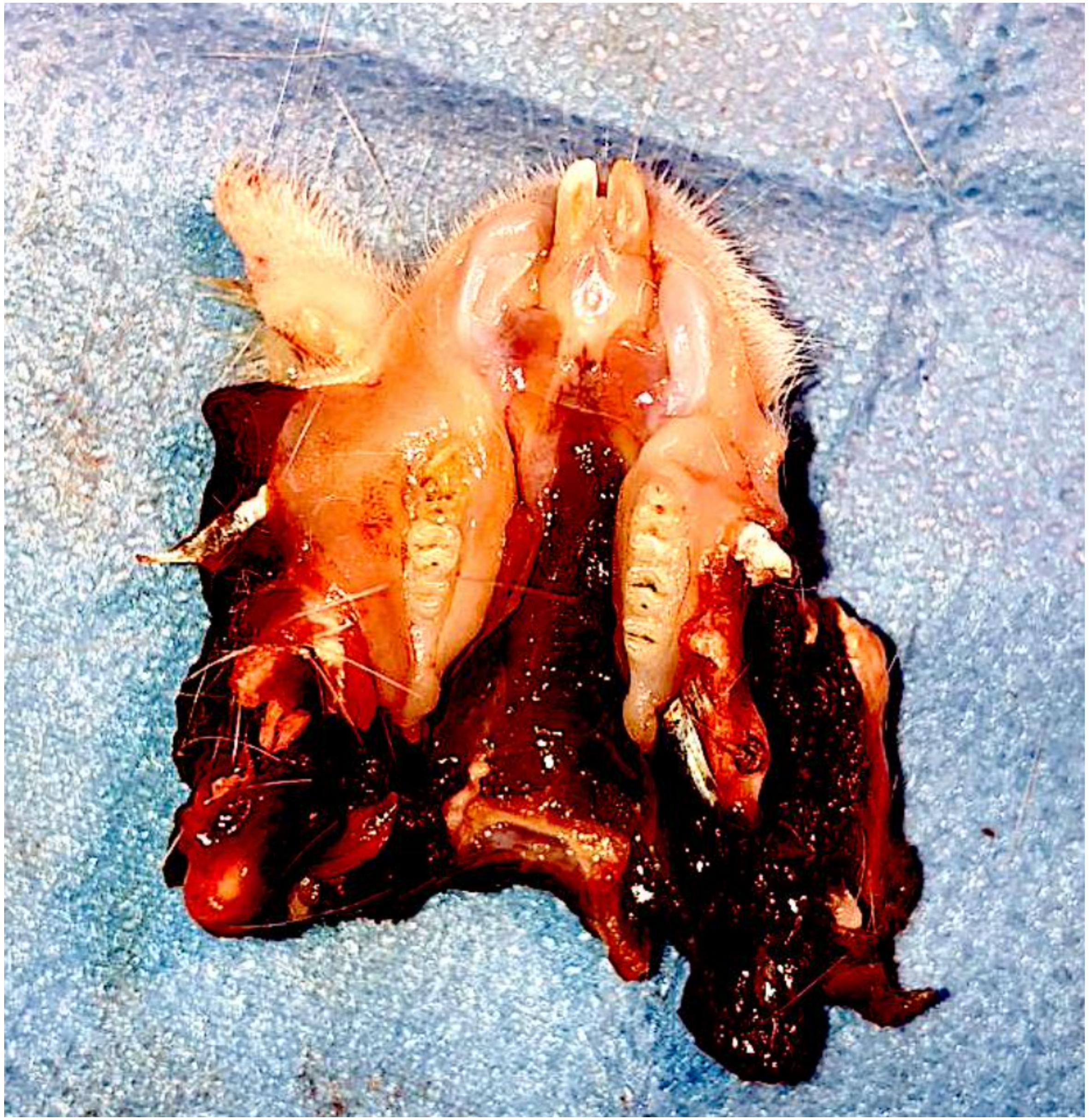

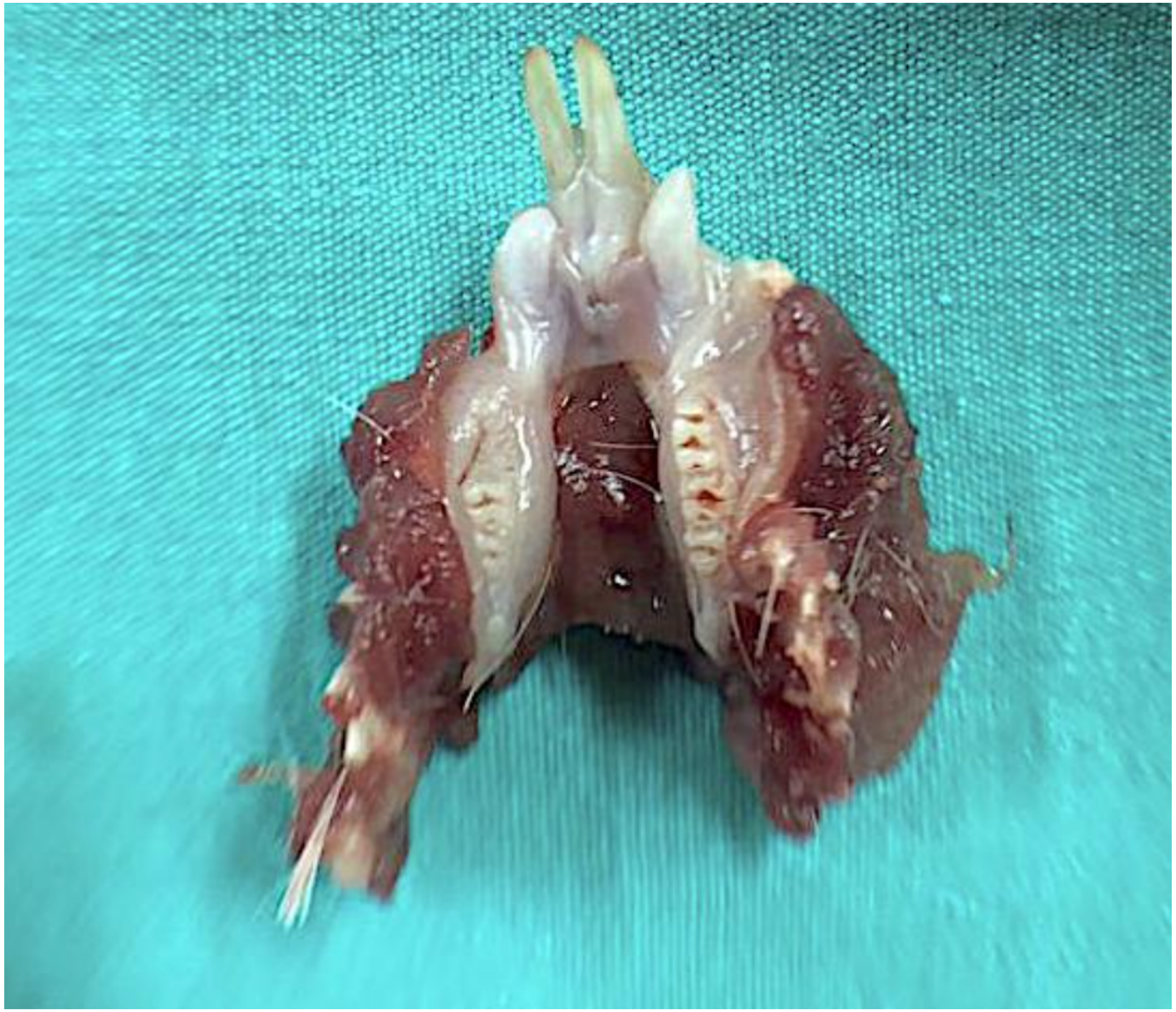

Supplement: Supplementary file 1 — Supplementary information. [file ijmsv21p2959s1.pdf]
